# UV-Polymerized Vinylimidazolium Ionic Liquids for Permselective Membranes

**DOI:** 10.3390/membranes10110308

**Published:** 2020-10-28

**Authors:** Fridolin O. Sommer, Jana-Sophie Appelt, Ingo Barke, Sylvia Speller, Udo Kragl

**Affiliations:** 1 Faculty of Interdisciplinary Research, Department Life, Light & Matter, University of Rostock, Albert-Einstein-Straße 25, 18059 Rostock, Germany; fridolin.sommer@uni-rostock.de (F.O.S.); ingo.barke@uni-rostock.de (I.B.); sylvia.speller@uni-rostock.de (S.S.); 2 Institute of Chemistry, University of Rostock, Albert-Einstein-Straße 3a, 18059 Rostock, Germany; jana-sophie.appelt@uni-rostock.de; 3 Institute of Physics, University of Rostock, Albert-Einstein-Straße 23, 18059 Rostock, Germany

**Keywords:** polymerized ionic liquids, polymeric membranes, membrane surface, membrane casting, nanofiltration

## Abstract

Ionic liquids are highly charged compounds with increasing applications in material science. A universal approach to synthesize free-standing, vinylalkylimidazolium bromide-containing membranes with an adjustable thickness is presented. By the variation of alkyl side chains, membrane characteristics such as flux and mechanical properties can be adjusted. The simultaneous use of different ionic liquids (ILs) in the synthesis can also improve the membrane properties. In separation application, these charged materials allowed us to retain charged sugars, such as calcium gluconate, by up to 95%, while similar neutral compounds such as glucose passed the membrane. An analysis of the surface conditions using atomic force microscopy (AFM) confirmed the experimental data and explains the decreasing permeance and increased retention of the charged sugars.

## 1. Introduction

Pure salts with a melting point below 100 °C, so-called “ionic liquids” (ILs), have remarkable properties such as low vapor pressure and non-flammability [1,2,3]. Due to their synthetic origin, a large number of combinations of anions and cations is possible, as well as the implementation of functional groups [4]. Commonly used structures of cations and anions of ILs are imidazolium, pyrrolidinium, phosphonium and halogenide ions [5].

Polymerizable groups, such as vinyl functions, enable one to fix the charges of ionic liquids in materials [6]. These might be hydrogels [7,8,9,10], coatings [11,12], conductive polymers [13,14,15] or polymeric films [16,17], whereby the synthetic route is not restricted to free radical polymerization and can be extended to other polymerization reactions or even the functionalization of uncharged materials [2,3,4,18,19,20]. Recently, Maksym et al. reported the influences in the synthesis of polyelectrolytes by the choice of reaction type. Different states of the material, thermal properties or conductivity are not only dependent on the choice of monomers or crosslinkers [21] but also on the synthetic strategy [15,22,23]. Besides, these IL materials can be used as catalysts themselves, whereas the process benefits from easy substrate removal [24]. In biomedical applications, hydrogels made of ionic liquids showed no leaching of toxic residuals after the polymerization [25] and in contact with animal cells; on the other hand, interesting antibacterial properties were observed [26]. The toxicity, as well environmental behavior, strongly depends on the structure of the cation and anion [27]. The IL 1-ethyl-3-methyl-1H-imidazol-3-ium-acetate has been thoroughly tested and registered within REACH (*Registration, Evaluation, Authorization and Restriction of Chemicals*), and, therefore, a number of tox tests have been made [28].

Polymerized ionic liquids (PILs) are gaining more and more interest in membrane science as they combine the advantages of ILs with those of polymers [15,29]. Charged membranes potentially increase the fouling resistance and permeability of aqueous solutions [30]. Zheng et al. reported a positive effect on antimicrobial activities of ILs but were not able to transfer these properties into the corresponding PIL membranes [31]. In contrast, Sengupta et al. discovered a reduced number of colonies of *Staphylococcus aureus* and *Pseudomonas aeruginosa* while using micro- and ultra-filtration membranes consisting of imidazolium monomers [32].

Another factor that significantly influences the properties of PILs is the length of the vinylimidazolium alkyl chain and the halides. In general, thermal stability is decreased by increasing side chains, while, at the same time, the impact on the glass transition temperature *T*_g_ depends on the anion. Herein, the temperature declines with more complex anions from halogenates towards bistriflimides. Thus, thermal-resistant materials as polyelectrolytes for electroactive devices such as fuel cells or electrode coatings are feasible [16].

Vinylpyridinium PILs were used as an adsorbent for chromium compounds in acidic aqueous solutions [33]. Wieszczycka et al. reported similar structures that also retained the heavy metal ions Cd^II^, Zn^II^ and Cu^II^ especially at higher temperatures. The porous material could be regenerated without any losses in adsorption capacity. Regeneration steps were performed with aqueous desorbing solutions containing ethylenediaminetetraacetic, nitric or hydrochloric acid [34].

UV-induced polymerizations represent techniques to fabricate PIL membranes under mild conditions. Due to the high number of charges, these layers were tested as anion exchange membranes in fuel cells [35,36,37,38]. Simultaneously, possible antimicrobial and antibacterial properties are of interest for wastewater treatment or wound-healing [31,32,39,40].

In this work, we report the synthesis of vinylimidazolium-based polymerized ionic liquids as free-standing nanofiltration membranes. PIL membranes with varying length of the IL alkyl chains were characterized by their water permeance. Additionally, atomic force microscopy (AFM) was used to study the surfaces and structural composition of these layers. Alterations in the properties were investigated by the mixture of ILs with a differing length of alkyl chains in the formation process. Furthermore, separation experiments were performed to evaluate the retention of charged and neutral sugars, their long-term stability and performance.

## 2. Materials and Methods

### 2.1. Chemicals

All chemicals were, if not mentioned otherwise, used as purchased from Sigma-Aldrich (Steinheim, Germany), Acros Organics (Geel, Belgium), TCI (Tokyo, Japan), Merck (Darmstadt, Germany) and Alfa Aesar (Kandel, Germany). Technical grade ethanol and diethyl ether were purified by rotary evaporation before use. Ultrafiltration membranes were purchased from Amicon Bioseparations (Jaffrey, NH, USA).

### 2.2. Synthesis of Ionic Liquids (ILs)

The synthesis of ionic liquids is described in similar forms in the literature [7,11,16,41,42]. To synthesize vinylethylimidazolium bromide ([VEtIm]Br), vinylbutylimidazolium bromide ([VBuIm]Br), vinylhexylimidazolium bromide ([VHexIm]Br), and vinylbenzoilimidazolium bromide ([VBnIm]Br), a flame-dried flask equipped with a magnetic stirrer was filled under argon atmosphere with 1 equivalent of 1-vinylimidazole (VIm) and 1.25 equivalents of the corresponding bromide. The solution was purged with argon and stirred at room temperature for one hour. Then, the reaction mixture was allowed to react under argon atmosphere. After at least 72 h, the reaction solution was washed five times with diethyl ether to remove unreacted starting materials. The remaining solvents were removed by rotary evaporation and vacuum. 

[VEtIm]Br and [VBuIm]Br were obtained as solids, whereby [VEtIm]Br was obtained after 24 h of reaction. Both solids were purified in the same way as liquid products. To precipitate [VBuIm]Br, the already purified liquid was cooled down to −28 °C overnight, then further with liquid nitrogen. The frozen compound was washed quickly and remained solid afterward.

For the synthesis of vinyldodecane bromide ([VDodecIm]Br), an additional 250 mL·mol_VIm_^−1^ of purified EtOH were added before purging the reaction mixture. All other synthesis steps were performed in the same way.

The synthesis and purity of ionic liquids were verified by NMR and melting point measurements (see Supplementary Materials).

### 2.3. Formation of the PIL Layer

The general procedures were adapted from the literature [31,32]. They were performed with 20 mol% IL, 20 mol% styrene, 60 mol% ACN, 2 wt% crosslinker (divinyl benzene, DVB) and 5 wt% diphenyl(2,4,6-trimethylbenzoyl)phosphine oxide (TPO) as a photo initiator. The weight percentages are related to the mass of IL used in the experiment. When using several ILs in one experiment, weight percentages were related to the total mass of ILs in the experiment. Nevertheless, molar ratios between the other compounds were not changed.

All chemicals were mixed in a vial by shaking and in an ultrasonic bath for up to 45 min. For casting the layer, a film applicator (AB3407, TQC Germany, Hilden, Germany) and casting knife (TQC Germany and self-constructed, Rostock, Germany) were used. The casting proceeded onto clean and non-scratched glass or polyoxymethylene surfaces at a speed of 10 mm·s^−1^ and a thicknesses between 200 and 750 µm. After being irradiated for up to 30 min by one Osram Dulux^®^ S BL350 9W UV-A lamp (Osram, Munich, Germany), the layer was immersed for 18–24 h in ethanol and deionized water. To fit into the filtration cell, the layers were cut by a hole puncher or knife.

### 2.4. Analysis

NMR spectra were recorded onto a Bruker Avance 250 II, 300 III and 500 (Ettlingen, Germany) to control the purity of the ILs. *d*_6_-DMSO was used as a solvent if not mentioned otherwise.

Concentrations of calcium gluconate were determined by HPLC and internal calibration. HPLC was equipped with an RI detector K-2301, a degasser, a HPLC pump (all from Knauer, Berlin, Germany), a HyperREZ XP carbohydrate H+ 8 µm column (Phenomenex, Aschaffenburg, Germany), a Smartline autosampler 3800 and Eurochrom 2000 (both Knauer, Berlin, Germany) as analyzing software. An aqueous 0.005 mol∙L^−1^ H_2_SO_4_ eluent was used.

### 2.5. Experimental Setup

All measurements were performed using Schleicher&Schuell (70 mL, now: *Whatman*, London, UK) stirred dead-end filtration cells with a diameter of 43.53 mm and an effective membrane area of 14.88 cm^2^. Due to the glassy body of the cell, the pressure was limited to 6 bar. To ensure a homogenously distributed flow through the membrane and mechanical stability during the experiments, a polyethylene sinter plate (70 µm pore diameter, 1.6 mm strong, purchased from Reichelt Chemietechnik GmbH, Heidelberg, Germany) was installed as support for the membrane. The polymeric layer was inserted in a wet environment to avoid drying.

The flux was determined with deionized water. An initial 0.025 mol·L^−1^ sugar solution was used in separation experiments.

### 2.6. AFM Measurements

Small pieces were cut out of the membranes and glued on glass coverslips using epoxy resin for a rigid mount. AFM topographies were obtained with a commercial device (Park Systems NE100) in dynamic mode using metal-coated Si cantilevers (type ACTA, AppNano, Al-coating, 300 kHz and HA-HR, SpectrumInstruments, Au coating, 380 kHz). Setpoint and oscillation amplitude were chosen to minimize tip–sample interaction (soft tapping mode or non-contact mode). Several locations of the samples were investigated to ensure the significance of observations.

## 3. Results and Discussion

### 3.1. Formation of Polymeric Layers

For the synthesis of membranes with thin layers functioning as a selective barrier between two phases [43], different methods are reported [44,45,46,47], namely, phase inversion, sol–gel or extruding processes using organic polymers, ceramics or composite materials [43]. Membrane selectivity allows one or more substances to pass the barrier (permeate), while others are rejected (retentate or concentrate) [44].

**Figure 1:**
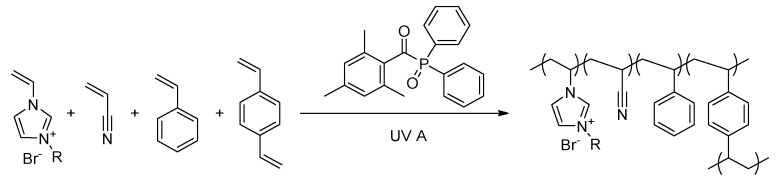
General polymerization reaction using diphenyl(2,4,6-trimethylbenzoyl)phosphine (TPO) as a UV photo initiator.

The synthesized membranes are flat sheets obtained by UV-induced polymerization of a casting solution containing the ionic liquid (IL), other monomers, a crosslinker (CL) and a photo initiator (PI) (Figure 1). Although benzoin ethyl ether (BEE) is reported as a suitable photo initiator [31,32,40,48,49], diphenyl(2,4,6-trimethylbenzoyl)phosphine (TPO) was nevertheless used as photo initiator in the following as it showed high reliability in reactivity when using different ILs.

Acrylonitrile (CAN) and styrene were used because they are known to provide polymeric layers with high chemical stability and improved mechanical properties [31,40,48]. They also served as solvents for solid and viscous ILs to provide a homogeneous casting solution. A glass plate served as a base, though it was not part of the following membrane process. Compared to materials like Teflon or poly(oxymethylene), the casted monomer film did not change its shape for hydrophilicity reasons and contracts.

All membranes were synthesized following the same procedure—only the IL was changed in the experiments. Experiments without the ionic liquids, using only the ratio of the other monomers, were not successful, as a solid layer was formed. Besides the IL, the photo initiator was also crucial to the reaction system.

**Figure 2:**
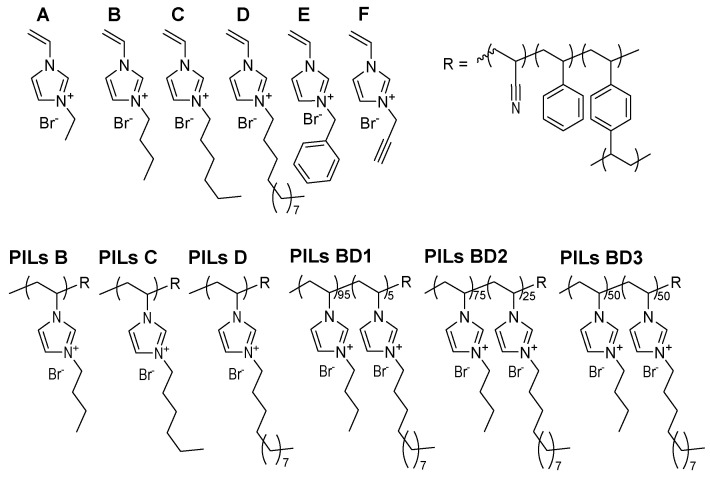
Ionic liquids used in membrane formation experiments and the corresponding polymers (A: [VEtIm]Br, B: [VBuIm]Br, C: [VHexIm]Br, D: [VDodecIm]Br, E: [VBnIm]Br, F: [VPrylIm]Br).

The ionic liquids used are shown in Figure 2. Contrary to the literature [31], a casting solution with A was possible by treating the solution eight hours in an ultrasonic bath. Hereby, the ultrasonic bath was heated up to 53 °C. However, the casted film did not react under UV irradiation and dissolved immediately and completely in the EtOH bath. No membrane was obtained.

More demanding imidazolium side chains, like in IL [VBnIm]Br (E) and [VPrylIm]Br (F), were challenging the reaction system. While F could not even be dissolved in the other monomers after eight hours of treatment in the ultrasonic bath and shaking, E always led to big holes in the resulting polymer layer.

Using IL [VBuIm]Br (B), [VHexIm]Br (C) and [VDodecIm]Br (D), a homogeneous PIL layer was obtained. The thickness chosen, adjustable from 1 to 3000 µm by the film applicator, was 300 µm. Thicker layers decreased the flux too much, whereas thinner layers were too unstable for the implementation in the filtration cell. By longer irradiation of thinner casted layers, the mechanical stability could not be increased. Nevertheless, it should be mentioned that the actual thickness after the reaction and washing is somewhat lower than 300 µm.

### 3.2. Flux Measurements

Water flux was determined by the volume of water passing at a certain time at different pressures. Every membrane fabricated as described above needed a certain conditioning time in the experimental setup. Besides the synthesized PIL membranes, commercial ultrafiltration (UF) and nanofiltration (NF) membranes were also characterized in the same manner to allow for comparison with those synthesized in this study. All flux data as a function of the pressure are shown in the supporting information (SI).

At first sight, using PIL membranes offers a broad range of flux rates by varying the side chain of the IL. Thus, the results demonstrate a linear behavior with small errors.

The application with PILs B with butyl side chains yielded in higher flux than a commercial 10 kDa UF membrane. A porous structure of the layer was conceivable. The main drawback is the lack of mechanical stability during the experiments. Compared to other PIL membranes, PIL B ruptures easily and responds sensitively to condition changes. 

To improve the usability, experiments with varying amounts of D were performed as the pure polymeric layer of D (PILs D) showed excellent mechanical stability. Unfortunately, the flux was one of the lowest observed and not quantifiable below 5 bar. While mixed PIL membranes were possible, the influence of D on the properties was more intense than the influence of B. PILs BD3, an equal molar mix of both ILs, decreased the flux significantly compared to PILs B and struggled with blocking. No flow was observed below 2.5 bar, and above this pressure the membrane blocked, prohibiting any flux.

Higher amounts of B improved the system. Both, PILs BD1 (B:D 20:1) and PILs BD2 (4:1) showed increased stability and usability. Even when the amount of D was lower, the flux decreased more than 180 times compared to PILs B and was only 13 times higher than PILs D. All mixed membranes showed lower flux than the commercial NF membrane under the same conditions. Table 1 gives an overview of the performance of PIL layers in the experiments, standardized to filtration area, time and pressure. The reproducibility of the PIL layers was also controlled by the flow.

In most of the common organic solvents (such as dimethylformamide, alcohols, *N*-methyl-2-pyrrolidone, chloroform, dichloromethane, hydrocarbons, pyridine, diethyl ether, dimethyl sulfoxide, acetone, and ethyl acetate), the PIL membranes were mechanically stable at room temperature. Fixed in the filtration setup, they showed the same stability. However, it was not possible to determine flux and related data. In some cases, the flux was fluctuating strongly even after long-time preconditioning; in other cases, the shape of the polymeric layer changed by deformation and was not usable anymore. Thus, the application of these membranes with organic solvents seems to be not possible.

### 3.3. Separation Experiments

After the characterization of the different PIL-containing layers, a separation experiment was conducted to determine the efficiency of the membranes. Calcium gluconate, a charged sugar derivate, was used as a test substrate in an aqueous solution.

A 0.025 mol·L^−1^ solution was used initially to avoid the precipitation of the sugar when overcoming the solubility limit (>0.069 mol·L^−1^). As the polymeric layers containing D monomer provide high mechanical stability, a mixture of 95 mol% B and 5 mol% D (PILs BD1) was used.

Overall, PILs BD1 was not suitable for the filtration of calcium gluconate, as the retention collapsed after 16 h (additional data and graphics in SI). The concentration of the sugar in the filtrate was increasing from the beginning. The permeance showed that after five hours, which could be ascribed to conditioning, a constant flow through the membrane was obtained. Furthermore, the concentrations and retention were constant after this time. More than 70% of the calcium gluconate was retained. Predictably, the concentration in the retentate increased over time. The permeance was 60% lower than in previous experiments. As the concentration in the filtrate increased significantly after twelve hours, a rupture of the membrane was assumed, which was confirmed by the inspection of the membrane after completing the experiment.

**Figure 3:**
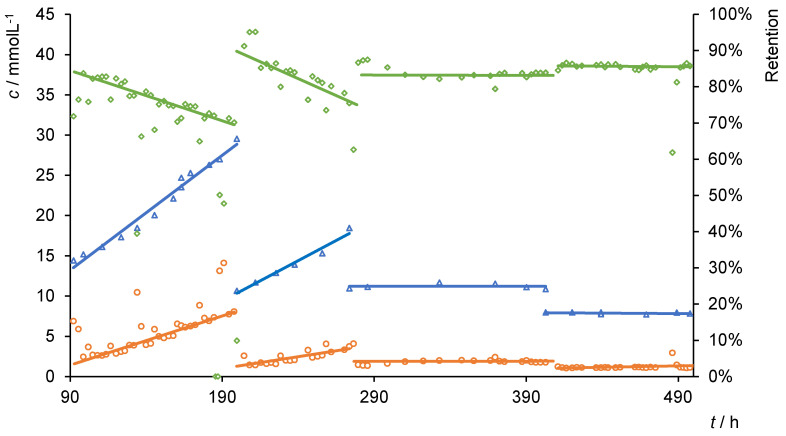
Retentate (blue triangles), filtrate (orange circles) and retention (green squares) in dead-end (90–270 h) and diafiltration experiments (270–490 h) using PILs BD2. Conditions: *d*_membrane_ = 43.35 mm, *p*_air_ = 6 bar, Schleicher&Schuell stirred dead-end cell, *n*_[VBuIm]Br_ = 0.00465 mol, *n*_[VDodecIm]Br_ = 0.00155 mol, *n*_IL_:*n*_ACN_:*n*_Styr_ = 1:3:1, 5wt% TPO, 2wt% divinyl benzene (DVB) (wt% based on *m*_IL_), 0.5 h ultrasonic bath, *h*_gap_ = 300 µm, 0.5 h UV lamp, 24 h EtOH, 24 h H_2_O.

When increasing the amount of D (PILs BD2) in the membrane formation, higher mechanical stability should affect the long-term stability positively. Figure 3 presents some of the results of the filtration experiments. The complete diagram, including all experimental data, is shown in the Supplementary Materials.

After a short time, in the beginning, the permeance was constant at approximately 0.05 L·m^−2^·h^−1^·bar^−1^. The retention *R* was about 75%. Compared to the pure water flux (Table 1), the permeance was reduced by more than 85% and displayed a stronger decrease than in other experiments. Within the filtration, the concentration *c*_sample_ increased slightly, while other parameters remained constant. This increase corresponds to the growing concentration *c*_cell_ in the filtration cell. It is assumed that a high concentration of calcium gluconate in the cell reduces the efficiency of the filtration process.

Originating from a lower concentration *c*_cell_ with 14.45 mol·L^−1^ (after 90 h), this observation was investigated more in detail. In over 100 h of filtration, both, the *c*_cell_ and *c*_sample_ increased equally. Higher retentate concentrations decreased the efficiency from about 84% to less than 40%. Here, the increase in calcium gluconate in the permeate seems to be linear and directly dependent on the concentration of the retentate. In addition, at high concentrations, the permeance is slightly decreased by about 10%.

The fluctuation in retention, concentration and permeance trace back to the experimental setup and an interruption overnight. The PIL-containing layer detached from the polyethylene plate, which was used as spacer. After establishing the filtration again, the same results as before were achieved (185–200 h). A continuous procedure overcame this drawback of the setup and confirmed the previous observations (205 to 280 h). Due to the low starting concentration of about 10.65 mol·L^−1^, retention of 95% was obtained. Nevertheless, the precedent linear enrichment of the sugar to 18.45 mol·L^−1^ reduced the retention to 74%.

Next, a steady concentration of approximately 11 mol·L^−1^ was used in a continuous experiment. Therefore, the volume of the filtrate was replenished to the cell in the form of pure water.

Between 270 and 400 operating hours, the concentration of calcium gluconate in the vessel was constant at 11.3 ± 0.3 mol·L^−1^. Simultaneously, the retention was above 83% and comparable to the retention obtained at the same concentrations in previous experiments. The permeance was unaffected by adding water at about 0.0535 L·m^−2^·h^−1^·bar^−1^. By lowering the concentration to 7.7 mmol·L^−1^, the retention increased to 87% and was constant. The retention is, as previously described, dependent on the retentate concentration. Lower retentate concentration results in higher rejections of calcium gluconate, while higher concentrations immediately impair the retention.

**Figure 4:**
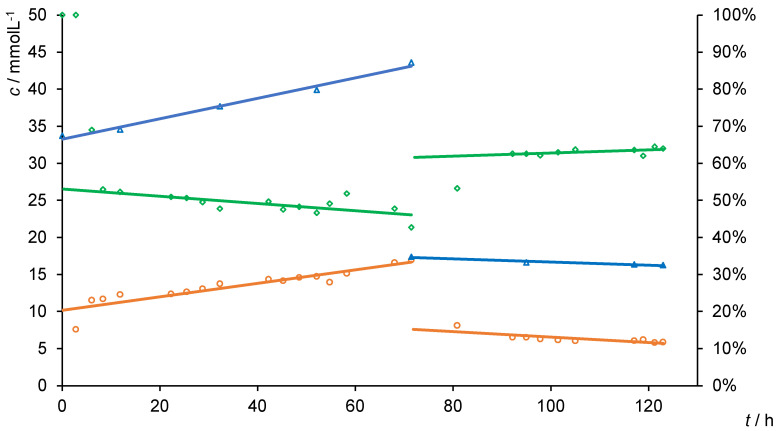
Retentate (blue triangle), filtrate (orange circles) and retention (green triangles) in dead-end (0–70 h) and diafiltration experiments (70–125 h) with sodium gluconate using PILs BD2. Conditions: *d*_membrane_ = 43.35 mm, *p*_air_ = 6 bar, Schleicher&Schuell stirred dead-end cell, *n*_[VBuIm]Br_ = 0.00465 mol, *n*_[VDodecIm]Br_ = 0.00155 mol, *n*_IL_:*n*_ACN_:*n*_Styr_ = 1:3:1, 5wt% TPO, 2wt% DVB (wt% based on *m*_IL_), 0.5 h ultrasonic bath, *h*_gap_ = 300 µm, 0.5 h UV lamp, 24 h EtOH, 24 h H_2_O.

Sodium gluconate provides a smaller molecular weight and the same structure as calcium gluconate. The experiment was started with a 0.025 mol·L^−1^ solution of sodium gluconate and the PIL BD2 membrane (Figure 4). The retention was about 50% higher for over 60 h with a permeance of 0.048 L·m^−2^·h^−1^·bar^−1^. Even with increased retentate concentration, no decreasing retention was observed when compared to that of the calcium gluconate (Figure 3). Equally, the retention was dependent on the initial feed concentration. By decreasing the initial feed concentration, the retention increased (90–120 h, Figure 4) and was only 20% lower than when using the calcium gluconate.

The comparison with neutral sugars showed that the charge has a significant influence on the separation performance. d-Glucose, d-mannitol, sucrose and raffinose were chosen because of their different structure and molecular weights. The retentions are summarized in Table 2. Further experimental details are presented in the SI. 

The retention increased from mono- to tri-saccharide to about 23%. This was much lower for the charged carbohydrates. Size exclusion was less pronounced than Donnan exclusion effects. Still, the gluconate salts were retained at different efficiencies (*R*_K+_ < *R*_Na+_ < *R*_Ca2+_). Similar behavior was reported by Cheng et al. using chloride salts [50]. The retention of the sugar salts conversed to the cation radius (*r*_Ca2+_ = 100 pm < *r*_Na+_ = 102 pm < *r*_K+_ = 138 pm) [51] but in agreement with the hydrate shell radius of these cations (*r*_h,K+_ = 331 pm < *r*_h,Na+_ = 358 pm < *r*_h,Ca2+_ = 412 pm) [52]. This suggests that the charge is essential for the efficient retention of substances, but these then differ in the radii of the hydrate shells and the associated charge density.

### 3.4. AFM Measurements

**Figure 5:**
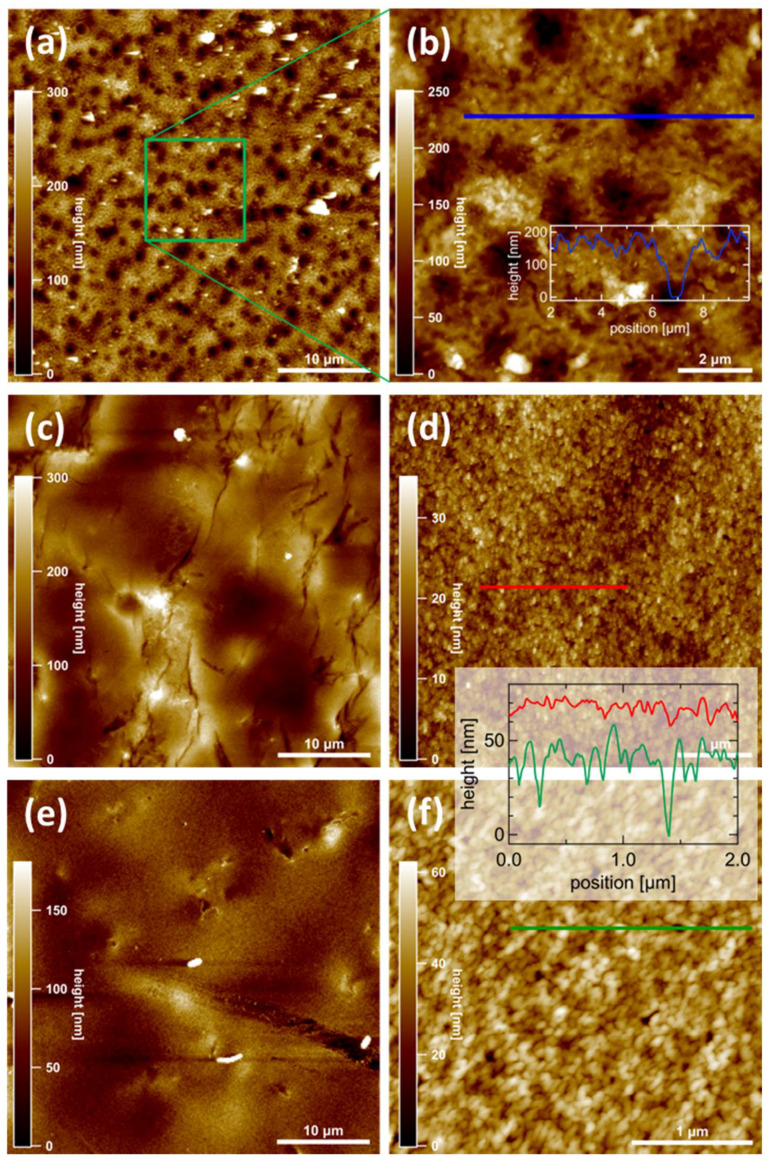
Morphology of membrane surfaces (at top sides) obtained by atomic force microscopy (AFM). Bright (dark) colors correspond to large (small) heights. The left column (**a**,**c**,**e**) contains overview images, while the right column (**b**,**d**,**f**) shows higher resolved images of the fine structure of three samples, i.e., (**a**,**b**) PILs B, (**c**,**d**) PILs BD2, (**e**,**f**) PILs D. Line profiles are shown as insets, taken at locations indicated by straight lines in the images (**b**,**d**,**f**).

Atomic force microscopy (AFM) was used to determine the membrane surface morphology on the micro- and nano-scale. Figure 5 summarizes the key observations for three samples, of which two were polymer layers with only one ionic liquid used in the formation process (PILs B and D) and one containing two ionic liquid monomers in the polymer (PILs BD2) after the filtration of pure water. All surfaces are rather flat on a larger scale (≥10 µm) with height corrugations of a very few 100 nm or below, but they exhibit different morphologic features on a smaller scale. On PILs B (Figure 5a,b), large depressions with a typical lateral size of about 1 µm are visible. Their apparent depth is about 150–200 nm, but this may be limited by the tip geometry of the AFM cantilever. Thus, these numbers represent a lower limit. Additionally, a grainy fine structure is visible with typical heights of 30–50 nm (Figure 5b). On PILs BD2 (Figure 5c), no pronounced holes are visible. Apart from a few grooves, no characteristic features are obvious on the microscale. On closer inspection (Figure 5d), again, a grainy structure is observed, but here the height corrugation is of the order of 10 nm or smaller. 

No pronounced holes are visible in the investigated area. At first glance, sample PILs D (Figure 5e) looks similar to PILs BD2, apart from the lower corrugation and a lower density of grooves. Even the appearance of the fine structure (Figure 5f) is similar to PILs BD2, but there are important differences: firstly, the corrugation height is considerably smaller, which is obvious from the comparison of line profiles (Figure 5d,f, inset); secondly, on PILs BD2, rather deep holes are visible (dark spots in Figure 5f and minima in the line profile, green curve) which are absent or at least much smaller on PILs D. Their depth cannot be directly quantified due to the tip geometry, but a lower limit is about 30 nm.

Nevertheless, different morphologies of PIL layers were pictured, and AFM measurements of the glass support excluded the possibility that the surface was shaped by the plate itself during the casting process.

Comparing AFM and flux data, an interesting correlation is found between the presence and size of surface holes and the measured permeance (compare Table 1): larger and more holes correspond to a higher flux. Thus, we tentatively assign the dominant liquid flux channels to the presence of hollow channels between the grains which are visible as holes in AFM topographies. This explains the observed flux data based on the membranes’ nanomorphology and provides a way to inspect and prescreen membranes without the need for elaborating flux measurements. However, the high density of the observed holes also results in less mechanically stable membranes with low retentions of charged sugars; thus, a mixture of IL monomers proved to be the optimal approach.

**Figure 6:**
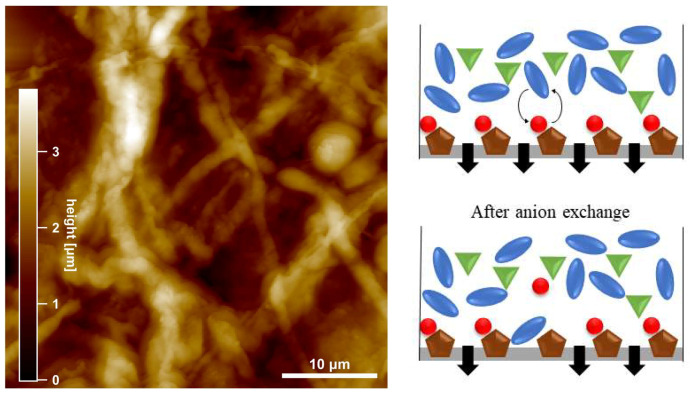
(**Left**) AFM topography of the top side of the membrane PILs BD2 after being used for filtration experiments with a calcium gluconate solution. Bright (dark) colors correspond to large (small) heights. Note the different height scale compared to Figure 5. (**Right**) Scheme of the possible anion exchange causing a decreased flux (brown: imidazolium cation, green: calcium cation, red: bromide, blue: gluconate).

Figure 6 shows the surface morphology of a membrane (PILs BD2) used for a filtration experiment of calcium gluconate similar to that shown in Figure 3. Large lengthy objects are visible which bear resemblance to fibers or fiber bundles. Their height is about a hundred times larger than the corresponding surface corrugation of the unused membrane (Figure 5c,d). Hence, this surface is presumably dominated by filtration residues which may have been formed by aggregation of molecules from the sugar solution.

Dot-like patterns with a similar appearance as in Figure 5 are well known to occur in systems undergoing pronounced microphase separations, such as block copolymers. This raises the question of whether the dark features may be due to phase separation of polymers in the PIL membrane. While we cannot rule out such behavior on the nanoscale, we note that topographic AFM under soft conditions as utilized here is much less sensitive on these features compared to, e.g., SEM reported recently elsewhere [53]. Due to the pronounced depth modulations in our case, we would like to rule out that the observed topography is an apparent one induced by the coexistence of different phases at the surface.

These deposits likely play a role in the time-dependent reduction of the flux through the membrane at different retentate concentrations (flux in Figure 3 compared to pure water, Table 1). Regarding the charged surface of the PIL membrane, an anion exchange between dissociated gluconate anions and bromide is feasible. This could lead to a decreased flux, as the larger gluconate ions on the membrane surface blocked channels and pores. The scheme in Figure 6 shows a possible exchange of the smaller coordinated bromide to the larger gluconate. Afterward, gluconate coordinated to the imidazolium cation and caused the blocking, which is also recognizable in the AFM and flux data. A decreasing flux due to concentration polarization is negligible, as the osmotic pressure when compared to the applied pressure is insignificant at these low concentrations. Additionally, a stirrer provided a homogeneous distribution of concentration as the flux did not decreases, even if the concentration was doubled.

## 4. Conclusions

In this work, the use of vinylimidazolium ionic liquids in polymeric membranes was investigated. The water permeance of these polymerized ionic liquids was investigated, and it was shown that the side chain of the ionic liquid monomer has a significant influence on the permeance. For the first time, mixtures of ILs were used in the membranes to benefit from the different physical and chemical advantages. The membranes used were mechanically stable for several hundred hours in the processes without changing their performances. In separation experiments, with the advantage of PIL charges, charged sugar derivates could be retained by up to 95%. Using AFM, a correlation between the efficiencies of different polymeric layers and their surface textures could be found. Moreover, residues of the charged sugars were observed and related to the decreasing flux in experiments. There is still a need to determine the final composition of the polymer film after being cast and irradiated, whereby the determination of the glass transition temperature *T*_g_ might provide an insight.

## Figures and Tables

**Table 1:** Overview of permeance *L*_H2O_ of different polymerized ionic liquid (PIL)-containing and commercial membranes.

Membrane	*L*_H2O_/L m^−2^ h^−1^ bar^−1^
Ultracell^®^ 30 kDa	193.4 ± 10.92
PILs B	74.09 ± 8.63
Ultracell^®^ 5 kDa	12.24 ± 0.57
PILs C	5.160 ± 0.53
DuraMem™ 200	1.880 ± 0.06
PILs D	0.027 ± 0.0021
PILs BD1	0.291 ± 0.0002
PILs BD2	0.259 ± 0.0041
PILs BD3	0.051 ± 0.0025 ^a^

Conditions: *d*_membrane_ = 43.53 mm, Schleicher&Schuell stirred dead-end cell, *n*_IL_ = 0.0031 mol, *n*_IL_:*n*_ACN_:*n*_Styr_ = 1:3:1, 5 wt% PI, 2 wt% CL (wt% based on *m*_IL_), 0.5 h ultrasonic bath, *h*_gap_ = 300 µm, 0.5 h UV lamp, 24 h EtOH, 24 h H_2_O. ^a^ Based only on results at 2.5 bar.

**Table 2:** Retention *R* of the sugars tested with PILs BD2.

Sugar	*M*/g∙mol^−1^	Charge	*R*
K-gluconate	234.25	yes	25%
Na-gluconate	218.14	yes	53%
Ca-gluconate	448.39	yes	75%
d-glucose	180.16	no	3%
d-mannitol	182.17	no	6%
Sucrose	342.30	no	14%
Raffinose	504.44	no	23%

Conditions: *c*_sugar_ = 0.025 mol∙L^-1^, *d*_membrane_ = 43.53 mm, *p*_air_ = 6 bar, Schleicher&Schuell stirred dead-end cell, *n*_IL_ = 0.0031 mol, *n*_IL_:*n*_ACN_:*n*_Styr_ = 1:3:1, 5 wt% PI, 2 wt% CL (wt% based on *m*_IL_), 0.5 h ultrasonic bath, *h*_gap_ = 300 µm, 0.5 h UV lamp, 24 h EtOH, 24 h H_2_O.

## Supplementary Materials

The following are available online at https://www.mdpi.com/2077-0375/10/11/308/s1, Figure S1: title, Table S1: title, Video S1: title. The synthesis and purity of ionic liquids were verified by NMR and melting point measurements. Figure S1. Pictures of (un-)successful layer formations with different; Figure S2. Aqueous flux measurement of synthesized and commercial membranes; Figure S1: Retentate, filtrate and retention in dead-end (0–270 h) and diafiltration experiments (270–590 h) with calcium gluconate using PILs BD2; Figure S4. Retentate, filtrate and retention in dead-end filtration with d-glucose using PILs BD2; Figure S5. Retentate, filtrate and retention in dead-end filtration with sucrose using PILs BD2; Figure S6. Retentate, filtrate and retention in dead-end filtration with raffinose using PILs BD2; Figure S7. Retentate, filtrate and retention in dead-end filtration with d-mannitol using PILs BD2.
