# Supplementary material for: UV-Polymerized Vinylimidazolium Ionic Liquids for Permselective Membranes
Source: Membranes (Basel). 2020 Oct 28;10(11):308. doi: 10.3390/membranes10110308 (PMC7692284; doi:10.3390/membranes10110308)
Supplement: Supplementary file 1 [file membranes-10-00308-s001.pdf]

membranes

Supplementary Materials

UV-Polymerized Vinylimidazolium Ionic Liquids for Permselective Membranes

Fridolin O. Sommer [1,2], Jana-Sophie Appelt [2], Ingo Barke [1,3], Sylvia Speller [1,3] and Udo Kragl [1,2,*]

[1] Faculty of Interdisciplinary Research, Department Life, Light & Matter, University of Rostock, Albert-Einstein-Straße 25, 18059 Rostock, Germany; fridolin.sommer@uni-rostock.de
[2] Institute of Chemistry, University of Rostock, Albert-Einstein-Straße 3a, 18059 Rostock, Germany; jana-sophie.appelt@uni-rostock.de
[3] Institute of Physics, University of Rostock, Albert-Einstein-Straße 23, 18059 Rostock, Germany; ingo.barke@uni-rostock.de (I.B.); sylvia.speller@uni-rostock.de (S.P.)
* Correspondence: udo.kragl@uni-rostock.de; Tel.: +49-381-498 6450

Contents

Pictures 2
NMR 3
[VEtIm]Br 3
[VBuIm]Br 5
[VHexIm]Br 7
[VDodecIm]Br 9
[VPryIm]Br 11
[VBnIm]Br 13
Flux Measurements 15
Filtration Experiments 15
Retention of Calcium Gluconate Monohydrate 16
Retention of D-Glucose 16
Retention of Sucrose 18
Retention of Raffinose 19
Retention of D-Mannitol 20

Pictures

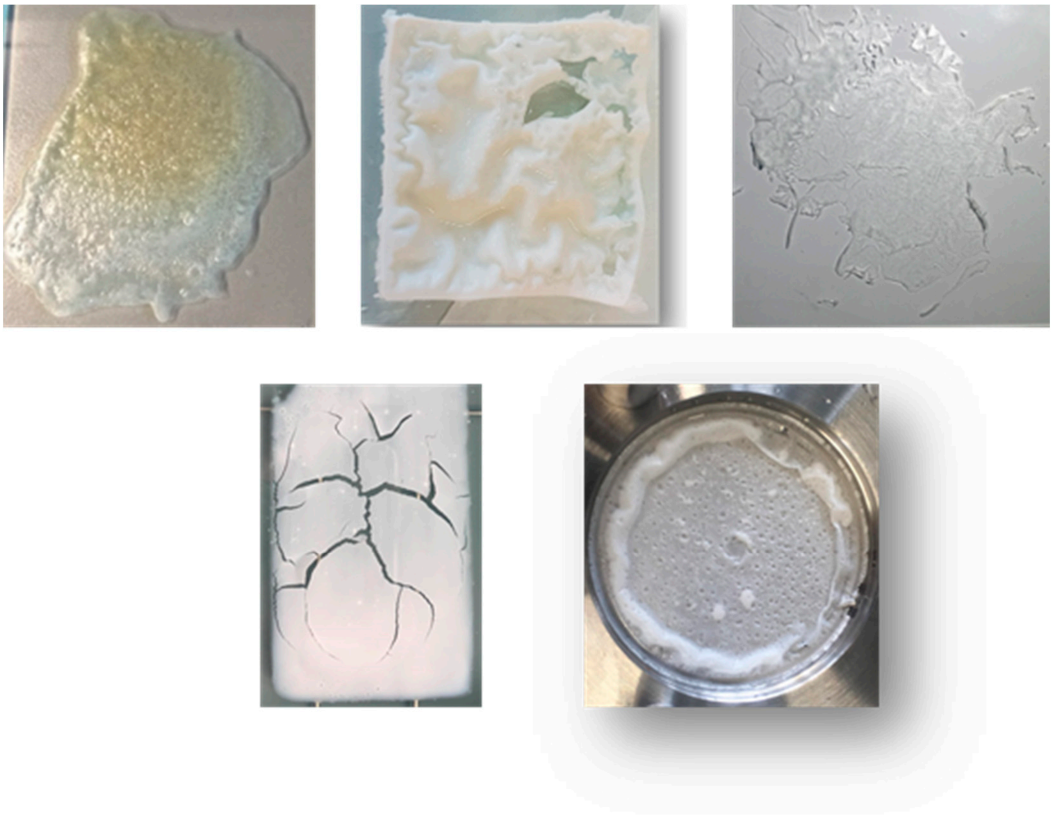

Figure S1. Pictures of (un-)successful layer formations with different ILs (I: too long irradiated, II: damage by wrong casting material, III: too thin, IV: dried after water bath, V: pores too big for application, VI: neat free standing PILs membrane).

NMR

[VEtIm]Br

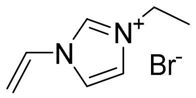

3-ethyl-1-vinyl-1-imidazole-3-ium bromide

$C_7H_{11}N_2Br$

M = 203.08 g·mol^{-1}

Calculated: C: 41.40 H: 5.46 N: 13.79

Measured: C: 41.740 H: 5.753 N: 13.957

$Y_{isol.}$ = 54%

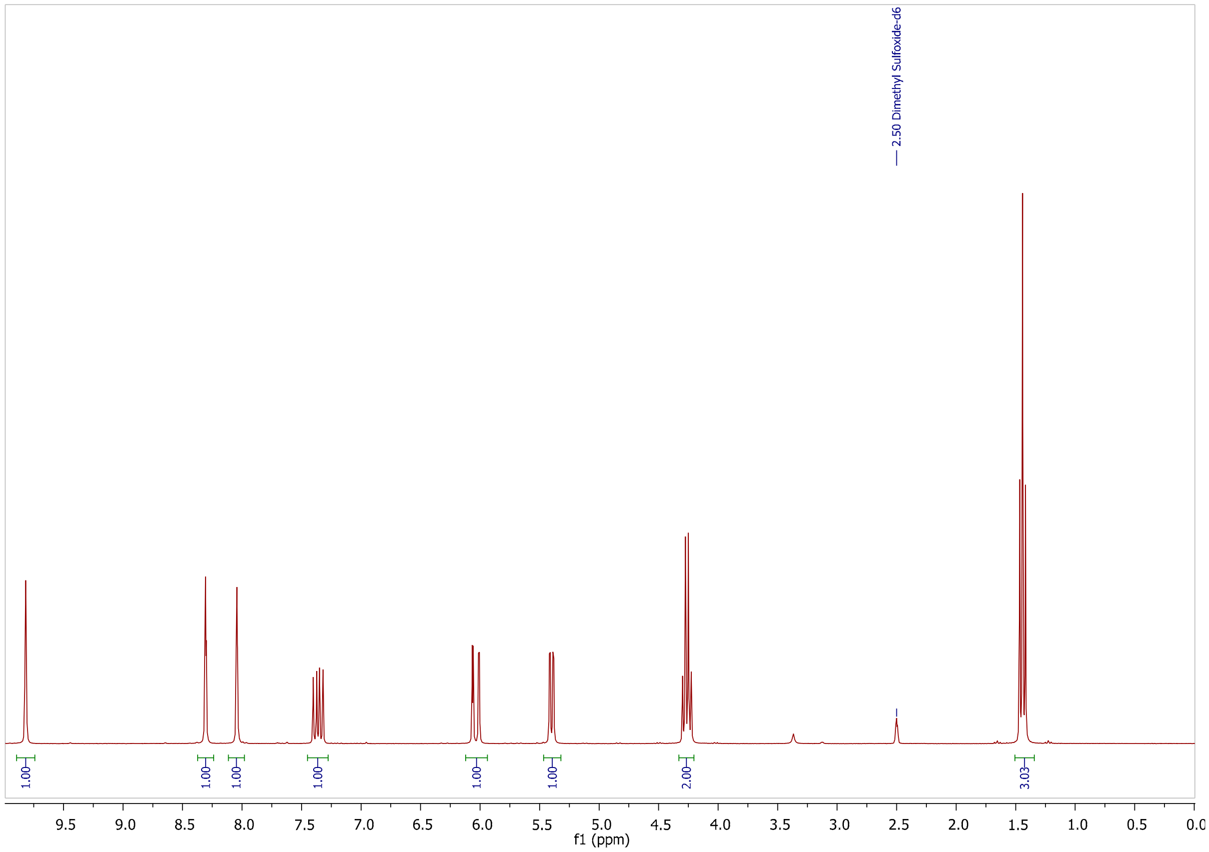

^{1}H NMR (300 MHz, DMSO) δ 9.82 (s, 1H), 8.31 (t, *J* = 1.8 Hz, 1H), 8.04 (t, *J* = 1.7 Hz, 1H), 7.36 (dd, *J* = 15.7, 8.8 Hz, 1H), 6.04 (dd, *J* = 15.7, 2.3 Hz, 1H), 5.40 (dd, *J* = 8.8, 2.3 Hz, 1H), 4.26 (q, *J* = 7.3 Hz, 2H), 1.44 (t, *J* = 7.3 Hz, 3H).

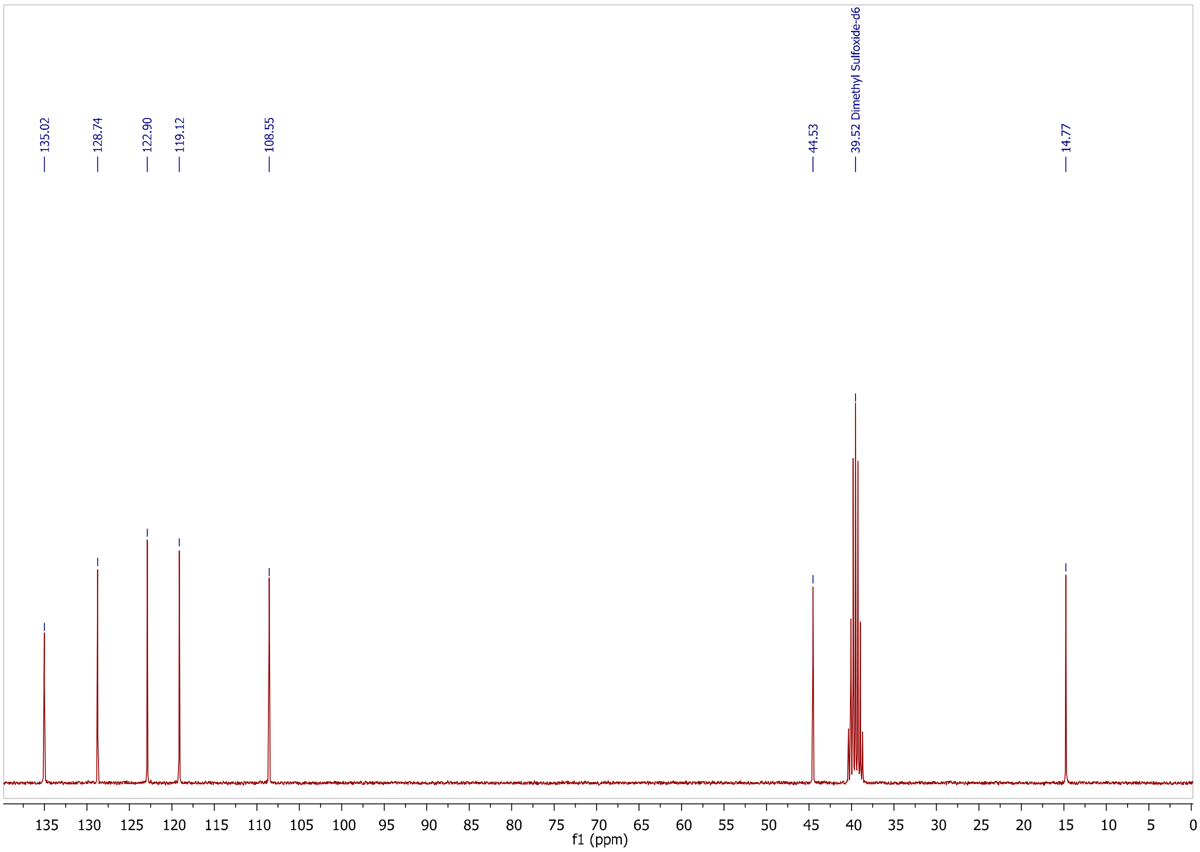

^{13}C NMR (75 MHz, DMSO) δ 135.02, 128.74, 122.90, 119.12, 108.55, 44.53, 14.77.

[VBuIm]Br

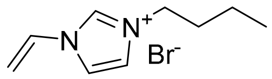

3-butyl-1-vinyl-1-imidazole-3-ium bromide

$C_9H_{15}N_2Br$

M = 231.14 g·mol^{-1}

Calculated: C: 46.77 H: 6.54 N: 12.12

Measured: C: 46.824 H: 6.438 N: 12.125

Melting point: 82 °C

$Y_{isol.}$ = 79%

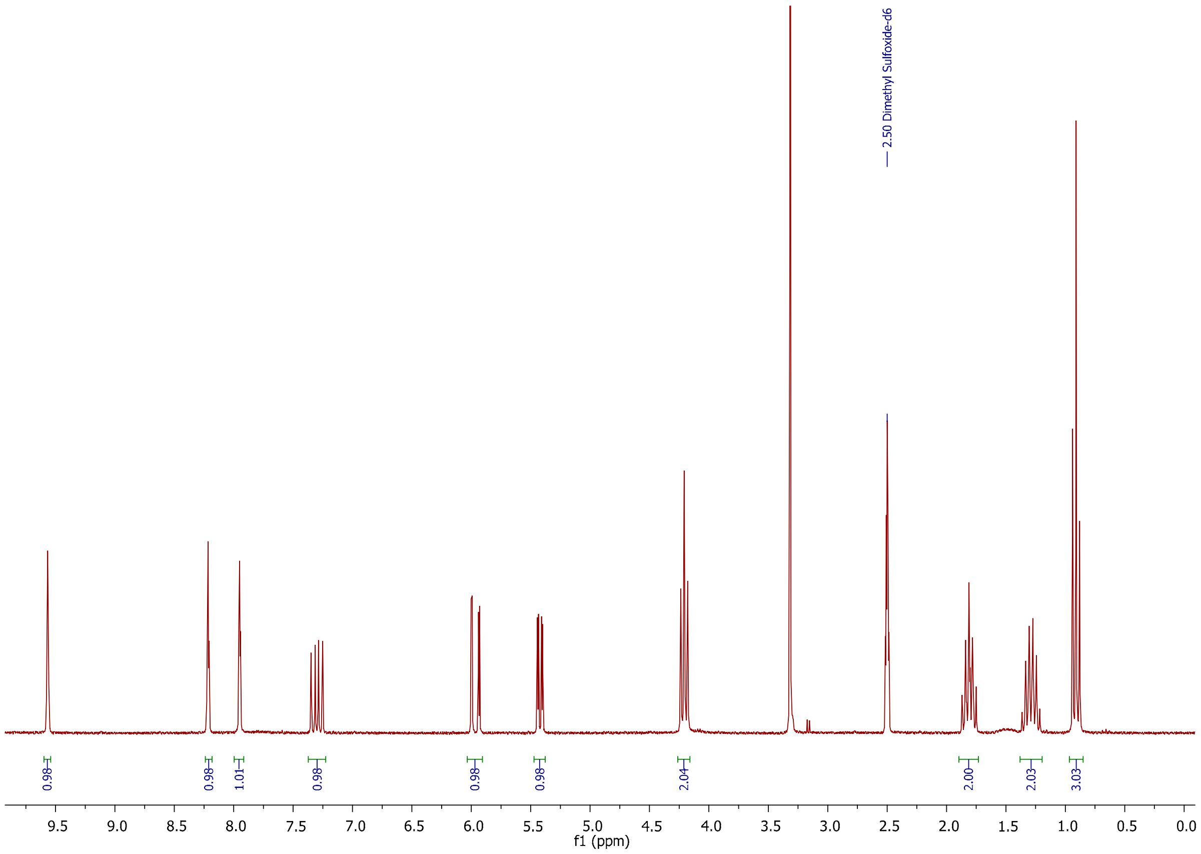

^{1}H NMR (250 MHz, DMSO) δ 8.21 (s, 1H), 7.94 (s, 1H), 7.25 (s, 1H), 5.93 (s, 1H), 5.40 (s, 1H), 4.19 (d, *J* = 7.1 Hz, 1H), 1.80 (s, 1H), 1.77 (d, *J* = 7.4 Hz, 1H), 1.26 (d, *J* = 7.3 Hz, 1H), 1.24 – 1.22 (m, 1H), 0.90 (d, *J* = 7.3 Hz, 1H).

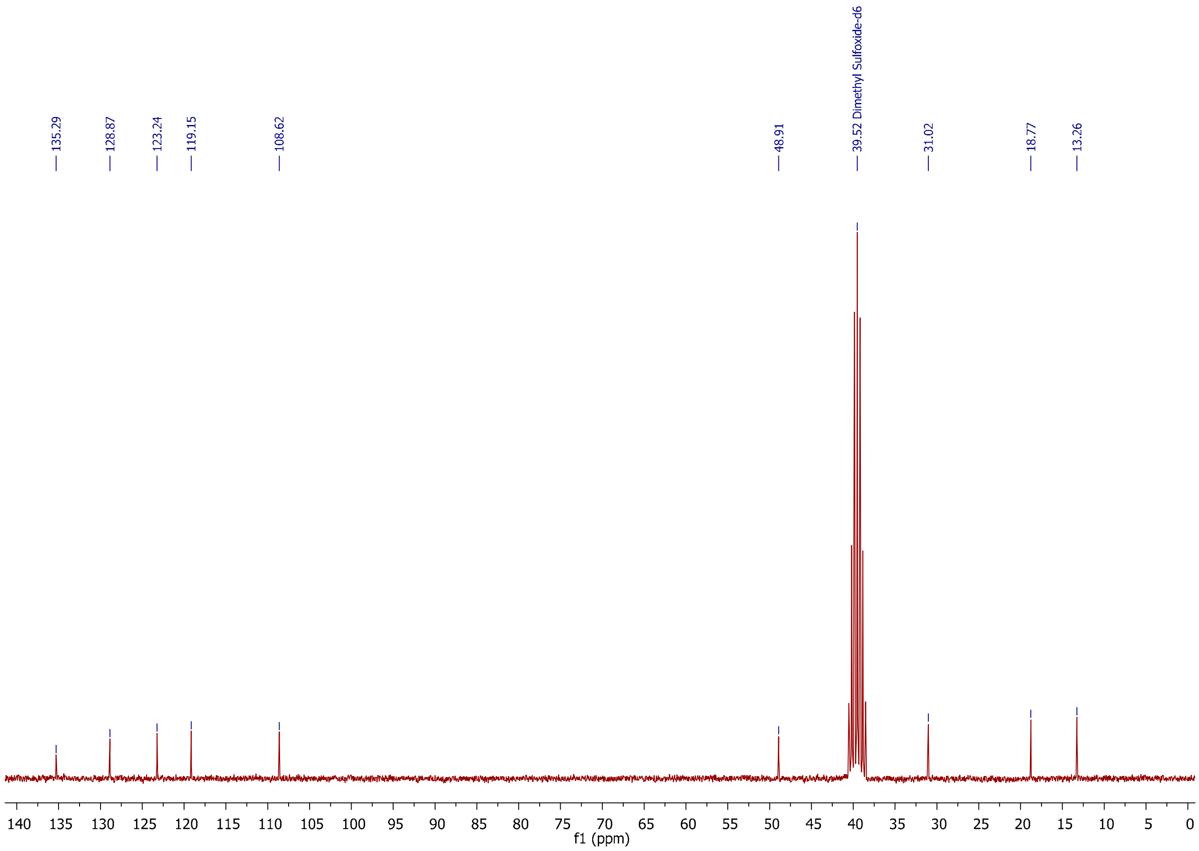

^{13}C NMR (63 MHz, DMSO) δ 135.29, 128.87, 123.24, 119.15, 108.62, 48.91, 31.02, 18.77, 13.26.

[VHexIm]Br

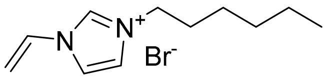

3-hexyl-1-vinyl-1-imidazole-3-ium bromide

$C_{11}H_{19}N_2Br$

M = 259.19 g·mol^{-1}

Calculated: C: 50.97 H: 7.39 N: 10.81

Measured: C: 51.048 H: 7.665 N: 11.113

Melting point: < 25 °C

$Y_{isol.}$ = 67%

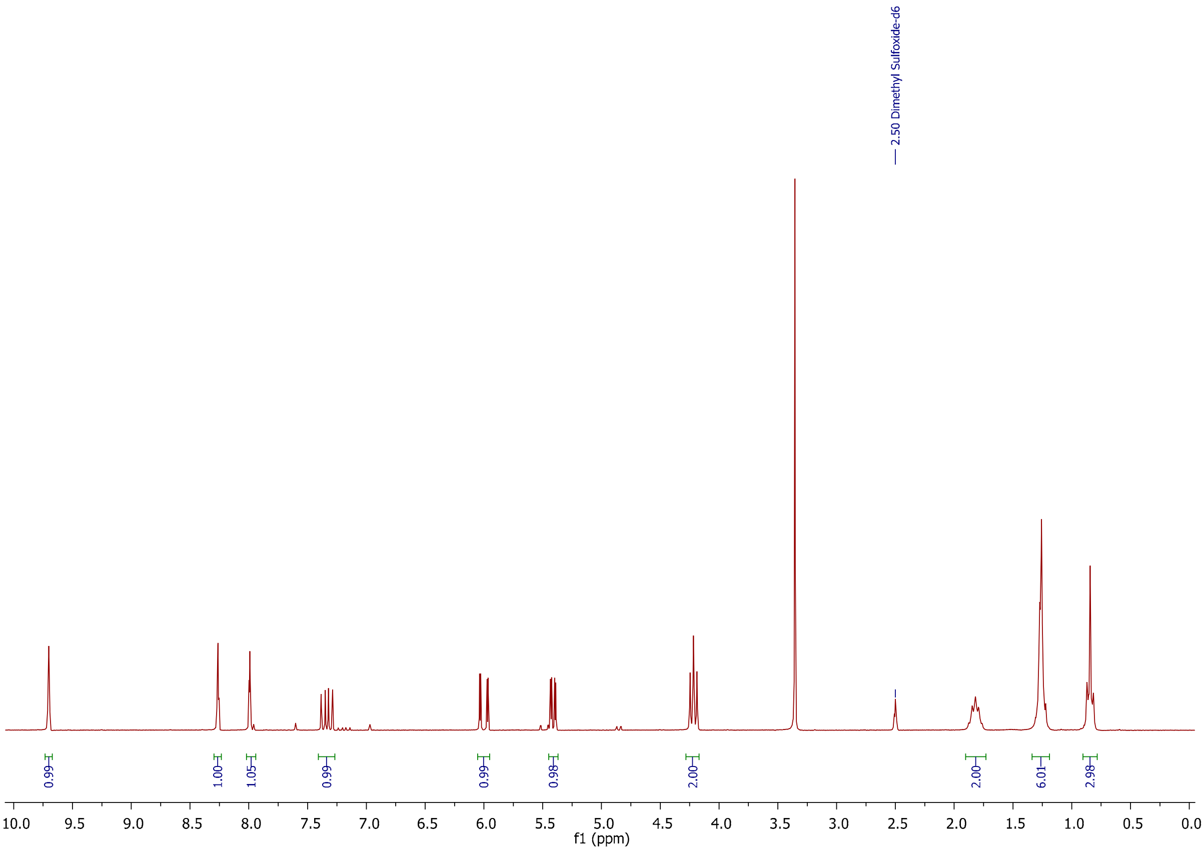

^{1}H NMR (250 MHz, DMSO) δ 9.70 (t, *J* = 1.5 Hz, 1H), 8.26 (t, *J* = 1.8 Hz, 1H), 7.98 (dd, *J* = 5.8, 4.1 Hz, 1H), 7.34 (dd, *J* = 15.7, 8.8 Hz, 1H), 6.00 (dd, *J* = 15.6, 2.4 Hz, 1H), 5.41 (dd, *J* = 8.7, 2.3 Hz, 1H), 4.22 (t, *J* = 7.3 Hz, 2H), 1.83 (dd, *J* = 13.8, 7.0 Hz, 2H), 1.34 – 1.19 (m, 6H), 0.90 – 0.78 (m, 3H).

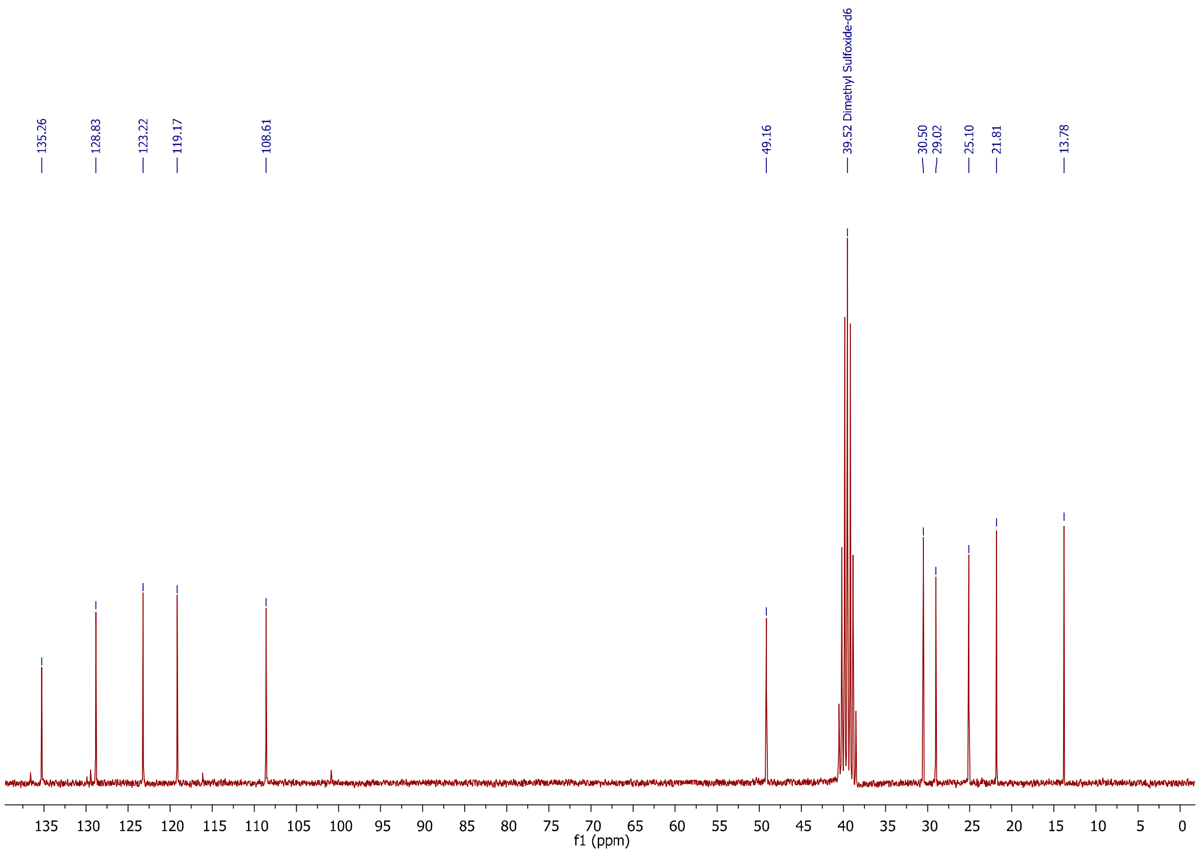

^{13}C NMR (63 MHz, DMSO) δ 135.26, 128.83, 123.22, 119.17, 108.61, 49.16, 30.50, 29.02, 25.10, 21.81, 13.78.

[VDodecIm]Br

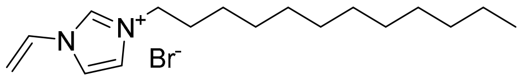

3-dodecyl-1-vinyl-1-imidazole-3-ium bromide

$C_{17}H_1N_2Br$

M = 343.35 g·mol^{-1}

Calculated: C: 59.47 H: 9.10 N: 8.16

Measured: C: 60.552 H: 9.257 N: 8.672

$Y_{isol.}$ = 87%

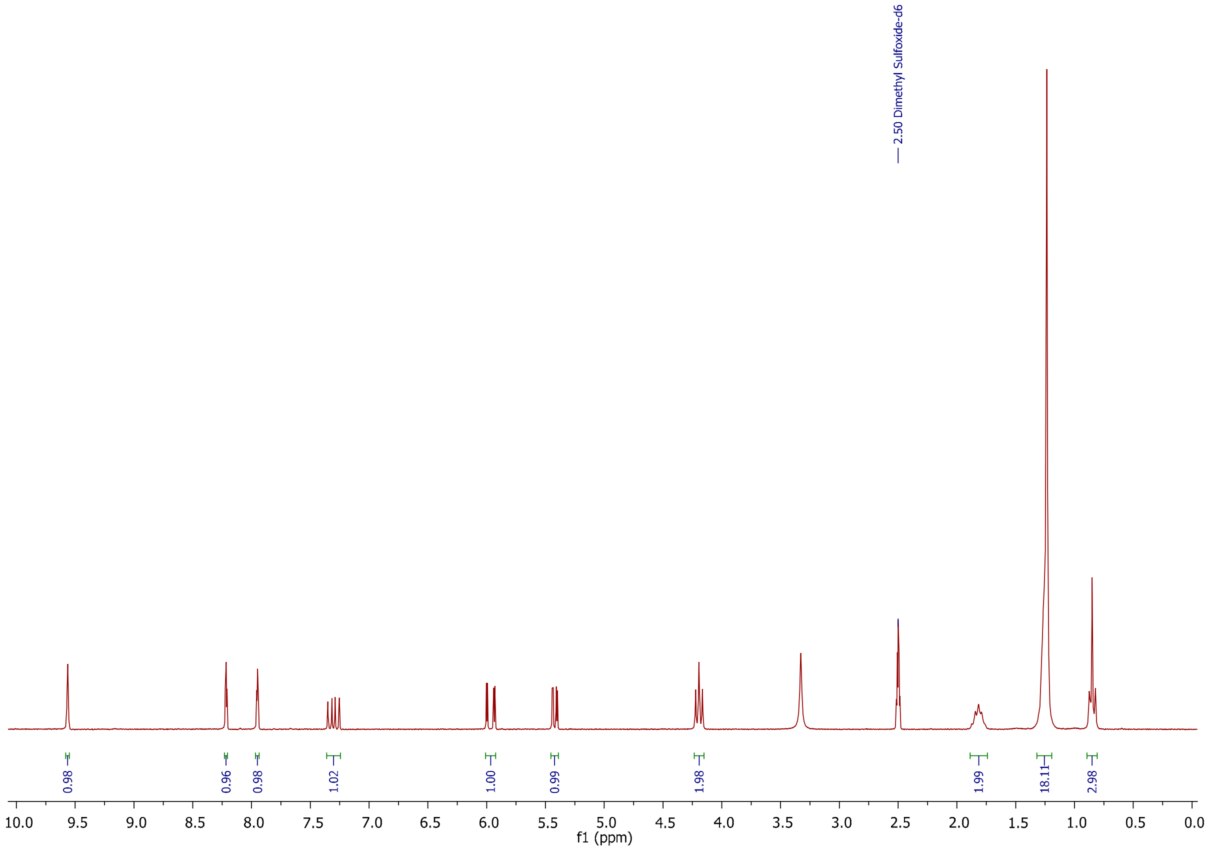

^{1}H NMR (250 MHz, DMSO) δ 9.56 (t, *J* = 1.5 Hz, 1H), 8.22 (t, *J* = 1.9 Hz, 1H), 7.95 (t, *J* = 1.7 Hz, 1H), 7.30 (dd, *J* = 15.6, 8.8 Hz, 1H), 5.97 (dd, *J* = 15.6, 2.3 Hz, 1H), 5.42 (dd, *J* = 8.7, 2.3 Hz, 1H), 4.19 (t, *J* = 7.2 Hz, 2H), 1.89 – 1.74 (m, 2H), 1.24 (s, 18H), 0.89 – 0.81 (m, 3H).

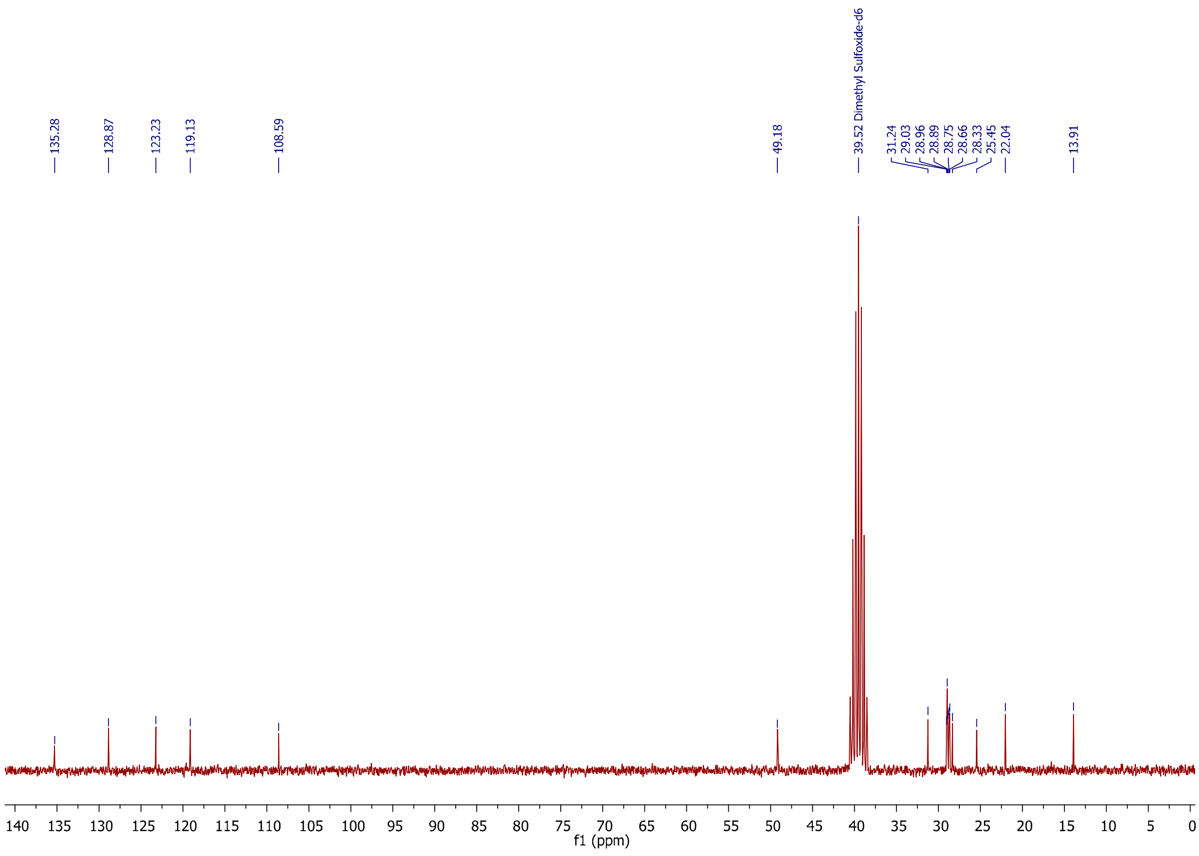

^{13}C NMR (63 MHz, DMSO) δ 135.28, 128.87, 123.23, 119.13, 108.59, 49.18, 31.24, 29.03, 28.96, 28.89, 28.75, 28.66, 28.33, 25.45, 22.04, 13.91.

[VPryIm]Br

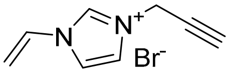

3-(prop-2-yn-1-yl)-1-vinyl-1-imidazole-3-ium bromide

$C_8H_9N_2Br$

M = 213.08 g·mol^{-1}

Calculated: C: 45.10 H: 4.26 N: 13.15

Measured: C: 45.15 H: 4.42 N: 13.29

$Y_{isol.}$ = 73%

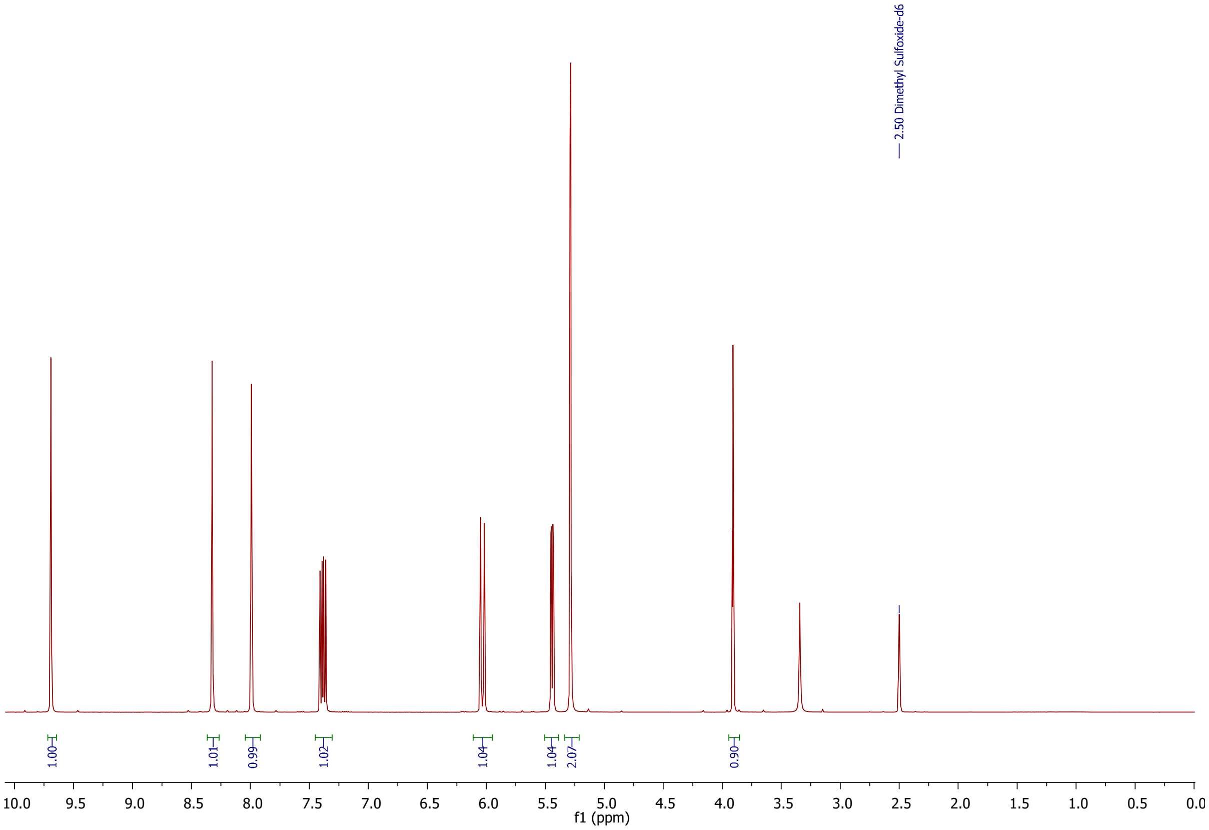

^{1}H NMR (500 MHz, DMSO) δ 9.69 (s, 1H), 8.32 (t, *J* = 1.7 Hz, 1H), 7.99 (s, 1H), 7.39 (dd, *J* = 15.7, 8.8 Hz, 1H), 6.03 (dd, *J* = 15.7, 2.4 Hz, 1H), 5.44 (dd, *J* = 8.7, 2.4 Hz, 1H), 5.29 (d, *J* = 2.5 Hz, 2H), 3.91 (t, *J* = 2.5 Hz, 1H).

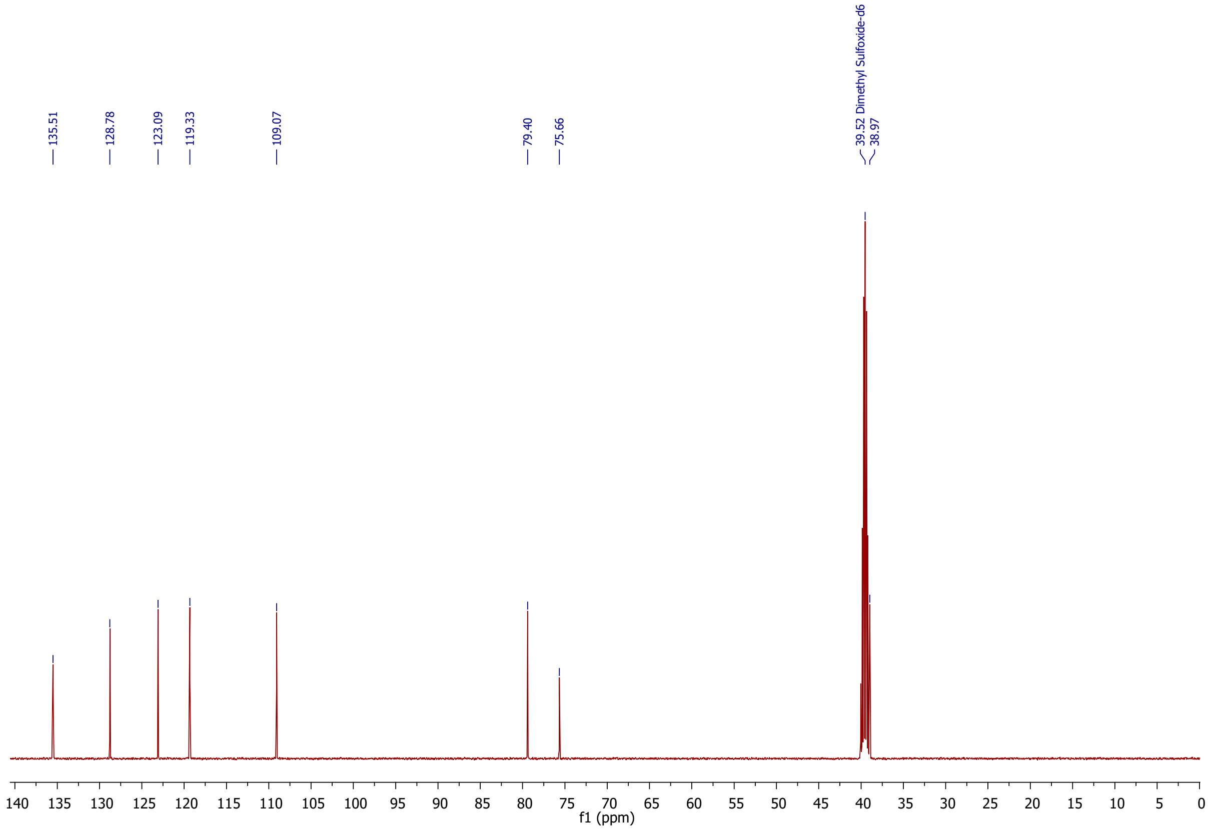

^{13}C NMR (126 MHz, DMSO) δ 135.51, 128.78, 123.09, 119.33, 109.07, 79.40, 75.66, 39.52, 38.97.

[VBnIm]Br

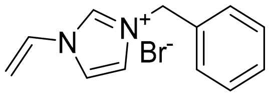

3-benzyl-1-vinyl-1-imidazole-3-ium bromide

$C_{12}H_{13}N_2Br$

M = 265.15 g·mol^{-1}

Calculated: C: 54.36 H: 4.94 N: 10.57

Measured: C: 54.464 H: 5.565 N: 10.050

$Y_{isol.}$ = 92%

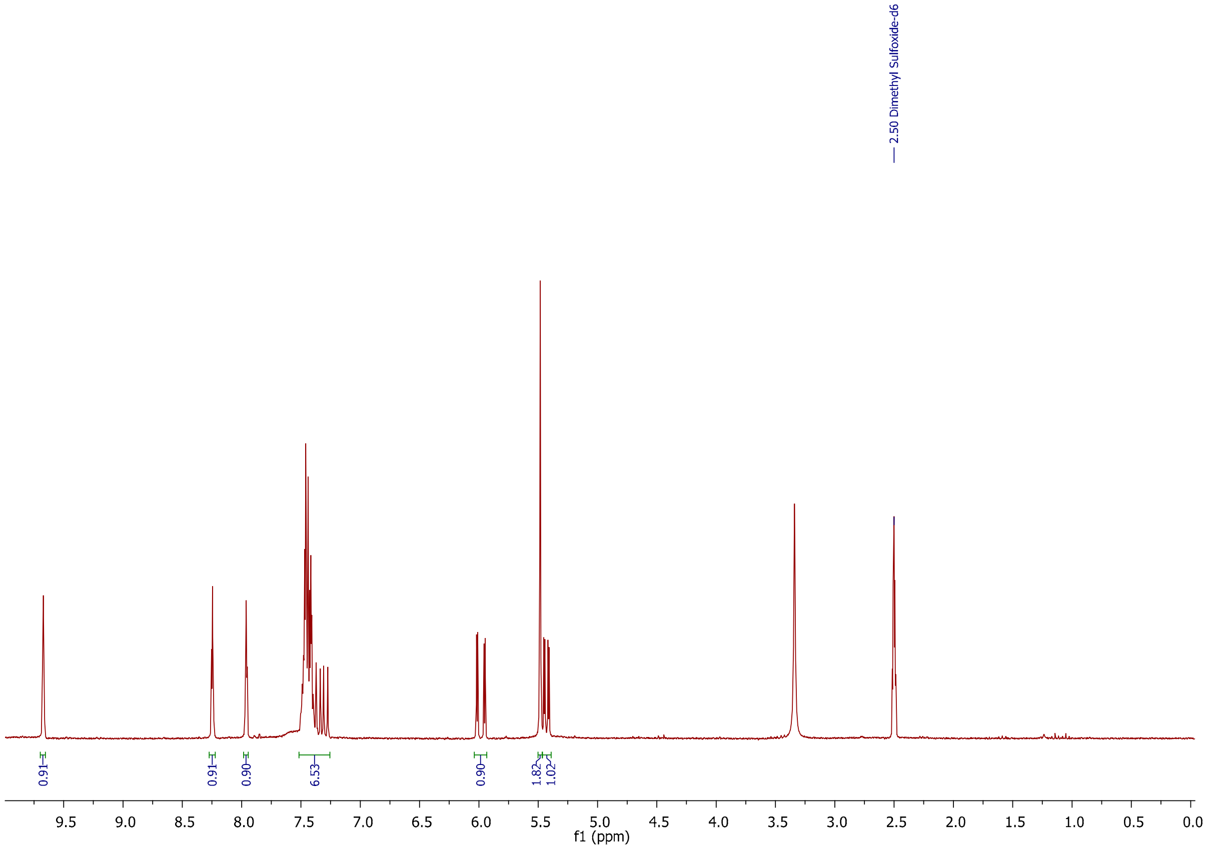

^{1}H NMR (250 MHz, DMSO) δ 9.67 (s, 1H), 8.25 (t, *J* = 1.8 Hz, 1H), 7.96 (t, *J* = 1.7 Hz, 1H), 7.52 – 7.26 (m, 7H), 5.98 (dd, *J* = 15.6, 2.4 Hz, 1H), 5.48 (s, 2H), 5.43 (dd, *J* = 8.7, 2.4 Hz, 1H).

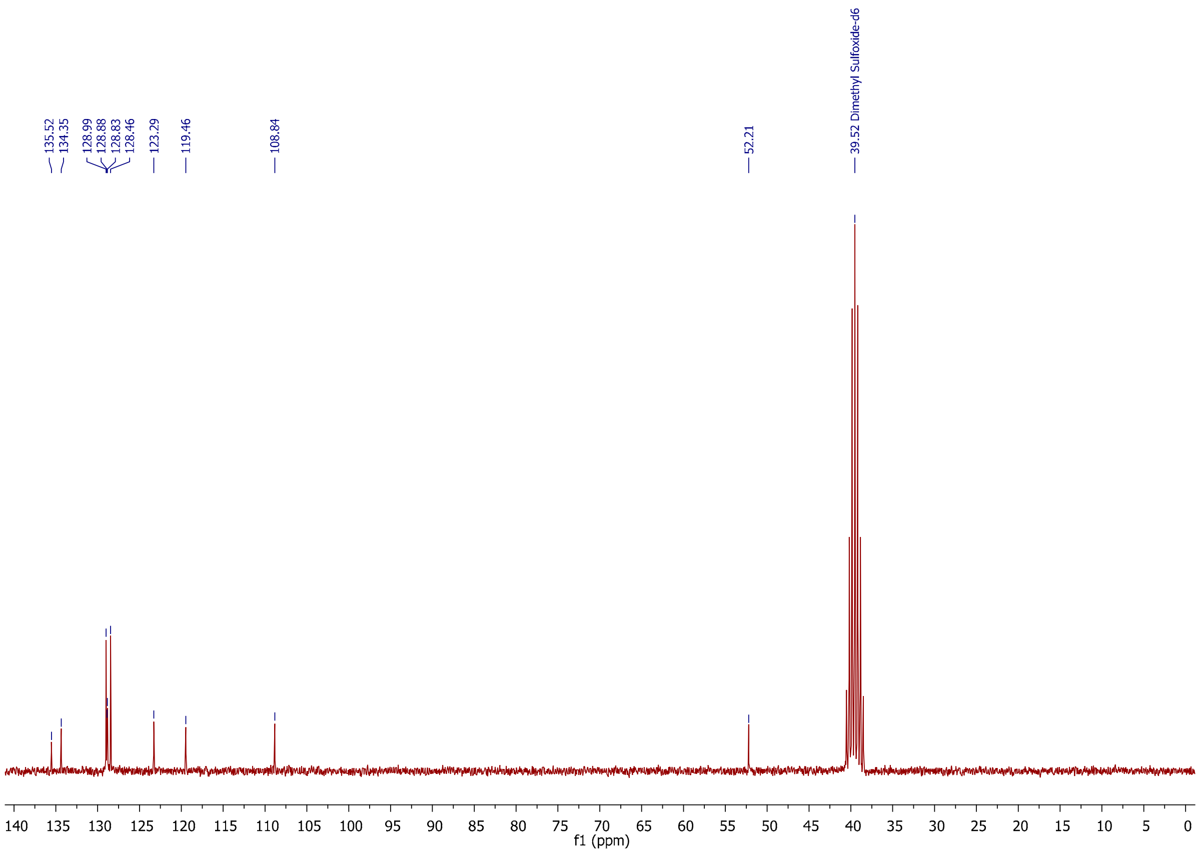

^{13}C NMR (63 MHz, DMSO) δ 135.52, 134.35, 128.99, 128.88, 128.83, 128.46, 123.29, 119.46, 108.84, 52.21.

Flux Measurements

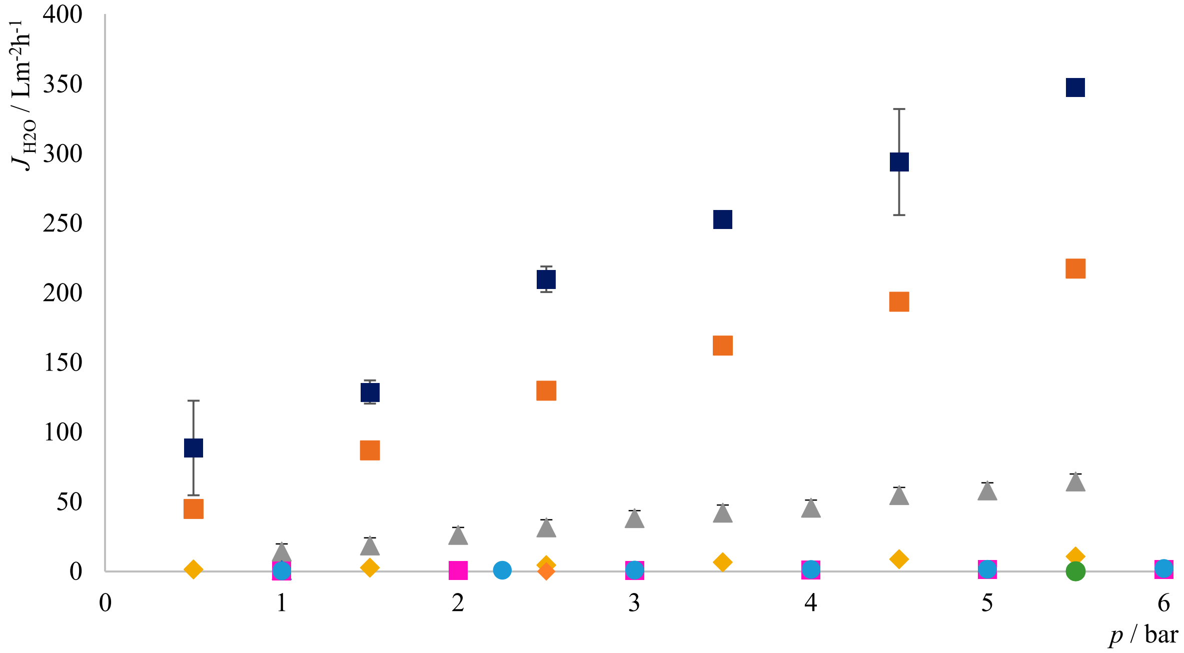

Figure S2. Aqueous flux measurement of synthesized and commercial membranes (Ultracell® 10 kDa, Ultracell® 5 kDa, DuraMem™ 200, **PILs B**, **PILs C**, **PILs D**, **PILs BD1**, **PILs BD2**, **PILs BD3**). Conditions: $d_{membrane}$ = 43.53 mm, p_{air} = 0.5 – 6 bar, Schleicher&Schuell stirred dead-end cell, n_{IL} = 0.0031 mol, n_{IL} : n_{ACN} : n_{Styr} = 1 : 3 : 1, 5 wt% PI, 2 wt% CL (wt% based on m_{IL}), 0.5 h ultrasonic bath, h_{gap} = 300 µm, 0.5 h UV lamp, 24 h EtOH, 24 h H_2O. Commercial membranes were used pre-conditioned as instructed by manufactures. Some error bars are small and covered by the data point.

Filtration Experiments

Retention of Calcium Gluconate Monohydrate

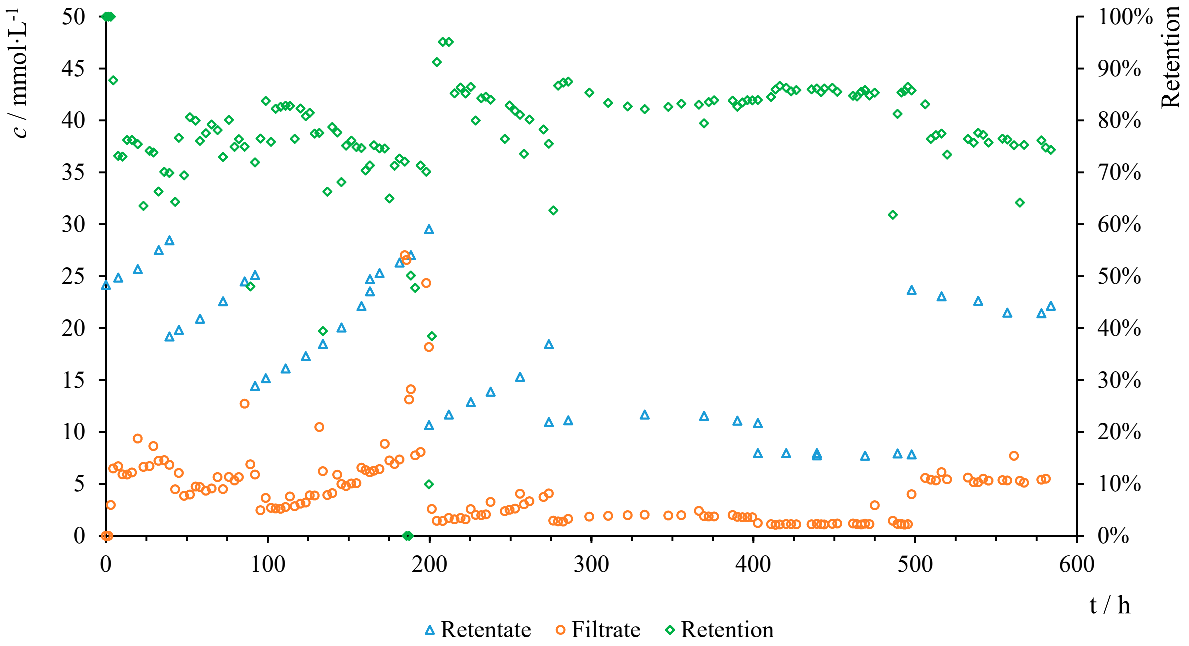

Figure S1: Retentate (blue), filtrate (orange) and retention (green) in dead-end (0–270 h) and diafiltration experiments (270–590 h) with calcium gluconate using PILs BD2. Conditions: $d_{membrane}$ = 43.35 mm, p_{air} = 6 bar, Schleicher&Schuell stirred dead-end cell, $n_{[VBuIm]Br}$ = 0.00465 mol, $n_{[VDodecIm]Br}$ = 0.00155 mol, $n_{IL} : n_{ACN} : n_{Styr}$ = 1 : 3 : 1, 5wt% TPO, 2wt% DVB (wt% based on m_{IL}), 0.5 h ultrasonic bath, h_{gap} = 300 µm, 0.5 h UV lamp, 24 h EtOH, 24 h H_2O.

Retention of D-Glucose

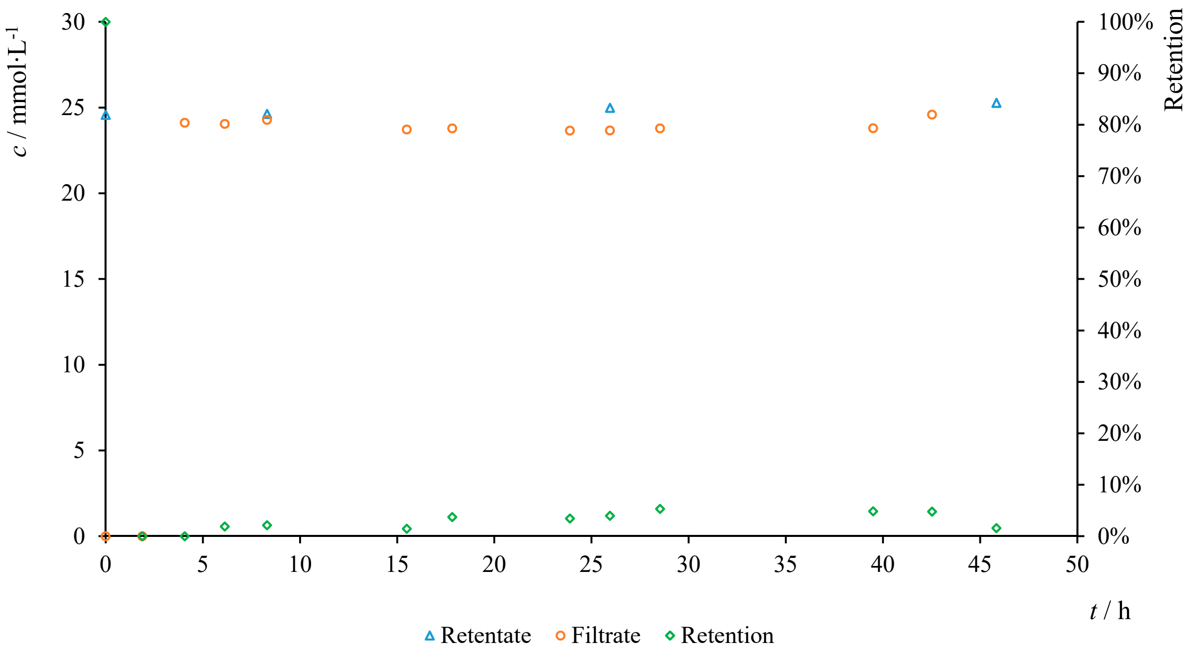

Figure S4. Retentate (blue), filtrate (orange) and retention (green) in dead-end filtration with D-glucose using PILs BD2. Conditions: $d_{membrane}$ = 43.35 mm, p_{air} = 6 bar, Schleicher&Schuell stirred dead-end cell, $n_{[VBuIm]Br}$ = 0.00465 mol, $n_{[VDodecIm]Br}$ = 0.00155 mol, n_{IL} : n_{ACN} : n_{Styr} = 1 : 3 : 1, 5wt% TPO, 2wt% DVB (wt% based on m_{IL}), 0.5 h ultrasonic bath, h_{gap} = 300 µm, 0.5 h UV lamp, 24 h EtOH, 24 h H_2O.

Retention of Sucrose

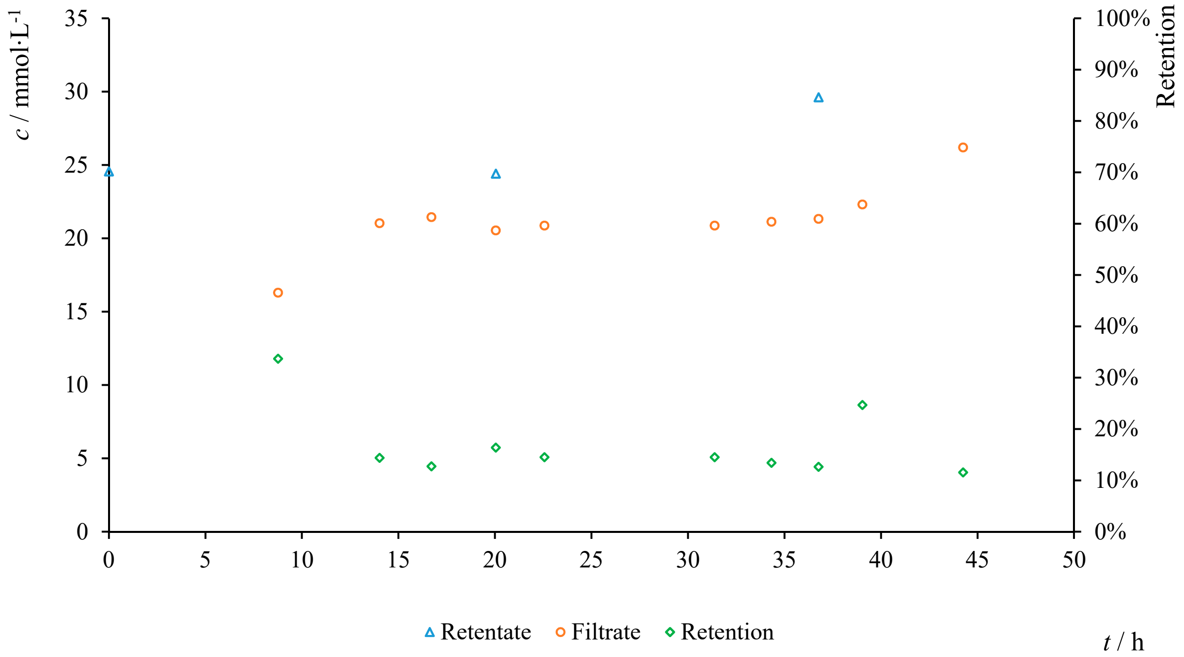

Figure S5. Retentate (blue), filtrate (orange) and retention (green) in dead-end filtration with sucrose using PILs BD2. Conditions: $d_{membrane}$ = 43.35 mm, p_{air} = 6 bar, Schleicher&Schuell stirred dead-end cell, $n_{[VBuIm]Br}$ = 0.00465 mol, $n_{[VDodecIm]Br}$ = 0.00155 mol, $n_{IL} : n_{ACN} : n_{Styr}$ = 1 : 3 : 1, 5wt% TPO, 2wt% DVB (wt% based on m_{IL}), 0.5 h ultrasonic bath, h_{gap} = 300 µm, 0.5 h UV lamp, 24 h EtOH, 24 h H_2O.

Retention of Raffinose

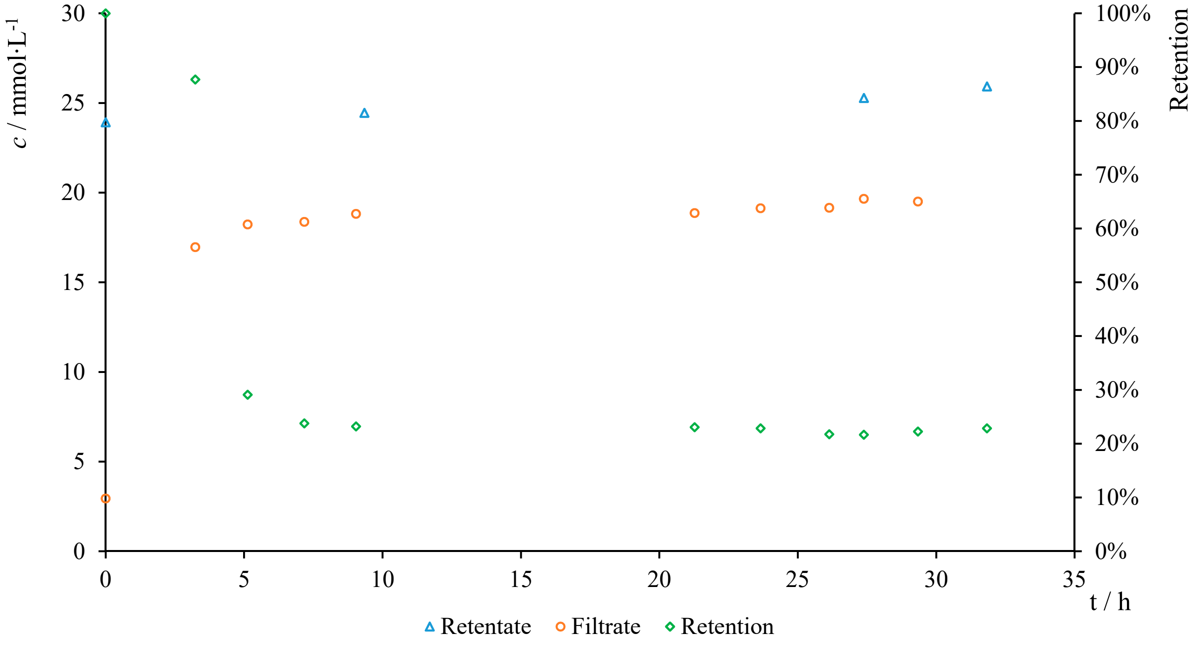

Figure S6. Retentate (blue), filtrate (orange) and retention (green) in dead-end filtration with raffinose using PILs BD2. Conditions: $d_{membrane}$ = 43.35 mm, p_{air} = 6 bar, Schleicher&Schuell stirred dead-end cell, $n_{[VBuIm]Br}$ = 0.00465 mol, $n_{[VDodecIm]Br}$ = 0.00155 mol, n_{IL} : n_{ACN} : n_{Styr} = 1 : 3 : 1, 5wt% TPO, 2wt% DVB (wt% based on m_{IL}), 0.5 h ultrasonic bath, h_{gap} = 300 µm, 0.5 h UV lamp, 24 h EtOH, 24 h H_2O.

Retention of D-Mannitol

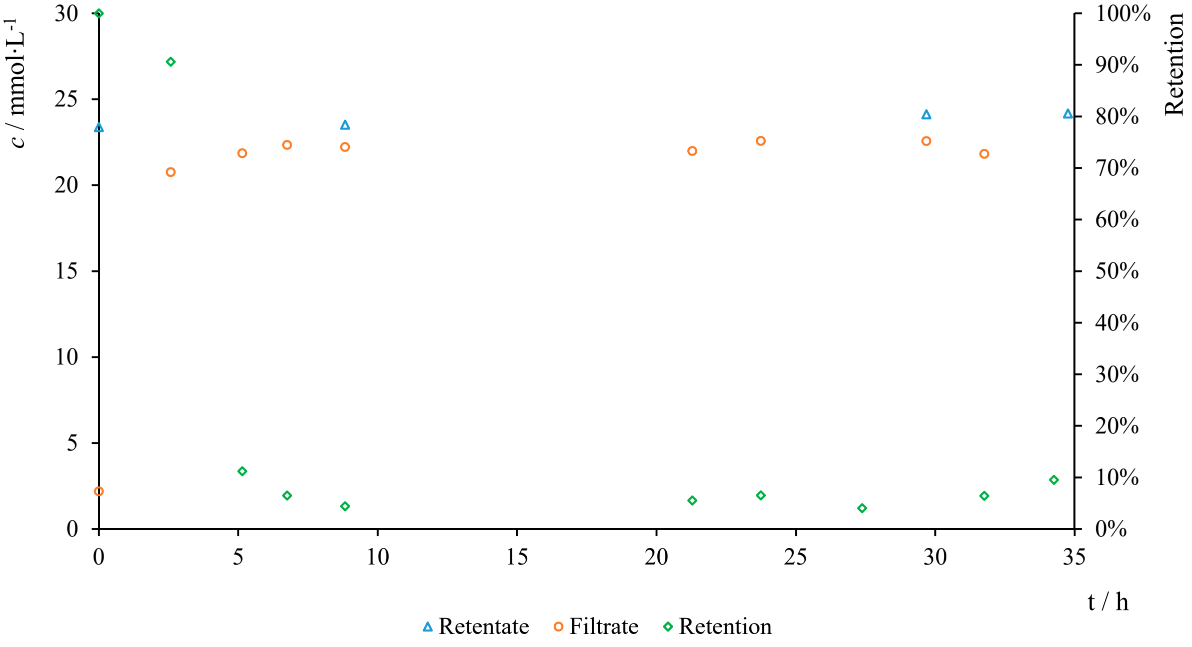

Figure S7. Retentate (blue), filtrate (orange) and retention (green) in dead-end filtration with D-mannitol using PILs BD2. Conditions: $d_{membrane}$ = 43.35 mm, p_{air} = 6 bar, Schleicher&Schuell stirred dead-end cell, $n_{[VBuIm]Br}$ = 0.00465 mol, $n_{[VDodecIm]Br}$ = 0.00155 mol, $n_{IL} : n_{ACN} : n_{Styr}$ = 1 : 3 : 1, 5wt% TPO, 2wt% DVB (wt% based on m_{IL}), 0.5 h ultrasonic bath, h_{gap} = 300 µm, 0.5 h UV lamp, 24 h EtOH, 24 h H_2O.
